# QTL Analysis of Dietary Obesity in C57BL/6byj X 129P3/J F_2_ Mice: Diet- and Sex-Dependent Effects

**DOI:** 10.1371/journal.pone.0068776

**Published:** 2013-07-29

**Authors:** Cailu Lin, Maria L. Theodorides, Amanda H. McDaniel, Michael G. Tordoff, Qinmin Zhang, Xia Li, Natalia Bosak, Alexander A. Bachmanov, Danielle R. Reed

**Affiliations:** Monell Chemical Senses Center, Philadelphia Pennsylvania, United States of America; German Institute for Human Nutrition, Germany

## Abstract

Obesity is a heritable trait caused by complex interactions between genes and environment, including diet. Gene-by-diet interactions are difficult to study in humans because the human diet is hard to control. Here, we used mice to study dietary obesity genes, by four methods. First, we bred 213 F_2_ mice from strains that are susceptible [C57BL/6ByJ (B6)] or resistant [129P3/J (129)] to dietary obesity. Percent body fat was assessed after mice ate low-energy diet and again after the same mice ate high-energy diet for 8 weeks. Linkage analyses identified QTLs associated with dietary obesity. Three methods were used to filter candidate genes within the QTL regions: (a) association mapping was conducted using >40 strains; (b) differential gene expression and (c) comparison of genomic DNA sequence, using two strains closely related to the progenitor strains from Experiment 1. The QTL effects depended on whether the mice were male or female or which diet they were recently fed. After feeding a low-energy diet, percent body fat was linked to chr 7 (LOD = 3.42). After feeding a high-energy diet, percent body fat was linked to chr 9 (*Obq5*; LOD = 3.88), chr 12 (*Obq34*; LOD = 3.88), and chr 17 (LOD = 4.56). The Chr 7 and 12 QTLs were sex dependent and all QTL were diet-dependent. The combination of filtering methods highlighted seven candidate genes within the QTL locus boundaries: *Crx*, *Dmpk*, *Ahr*, *Mrpl28*, *Glo1*, *Tubb5*, and *Mut*. However, these filtering methods have limitations so gene identification will require alternative strategies, such as the construction of congenics with very small donor regions.

## Introduction

Humans differ in their degree of obesity, and this is accounted for at least in part by their genetic constitution [1]. Differences among individuals can be accentuated when the usual diet is supplemented with additional energy [2], and this is also partially due to genotype [3]. Like humans, strains of rodents differ in their degree of obesity [4], and they also differ in their degree of weight gain when the energy density of their diet is altered [5], [6]. Unraveling the genetics of dietary obesity has become urgent because one cause of human obesity is thought to be the increasing availability of energy-dense food.

Gene-by-diet interactions are difficult to study in humans because the human diet is hard to control. Therefore, several animal models have been developed to study the genetics of obesity, and research is advancing to identify specific genes underlying these phenotypes [7], [8], [9], [10], [11], [12], [13], [14], [15]. One such model involves the C57BL/6ByJ (B6) and 129P3/J (129) inbred mouse strains. These inbred strains differ in their susceptibly to dietary obesity [16]. Previous work has demonstrated that a B6 x 129 F_2_ cross is a rich source of quantitative trait loci (QTLs) for obesity when mice are fed a relatively low-energy diet (chow) [17], [18], [19]. Here we examined the genetic basis of the response of this cross to a high-energy diet, by four methods. First, we used linkage analysis in F_2_ intercrossed mice derived from the B6 and 129 strains to find genetic loci that predispose these mice to develop dietary obesity. Then, because QTLs map to broad genomic regions, we used three further methods to identify candidate genes: genotype association mapping, gene expression, and sequence comparison. These methods have been useful in previous studies of complex phenotypes [14], [20], [21], [22], [23], [24], [25], [26].

## Materials and Methods

**Figure 1:**
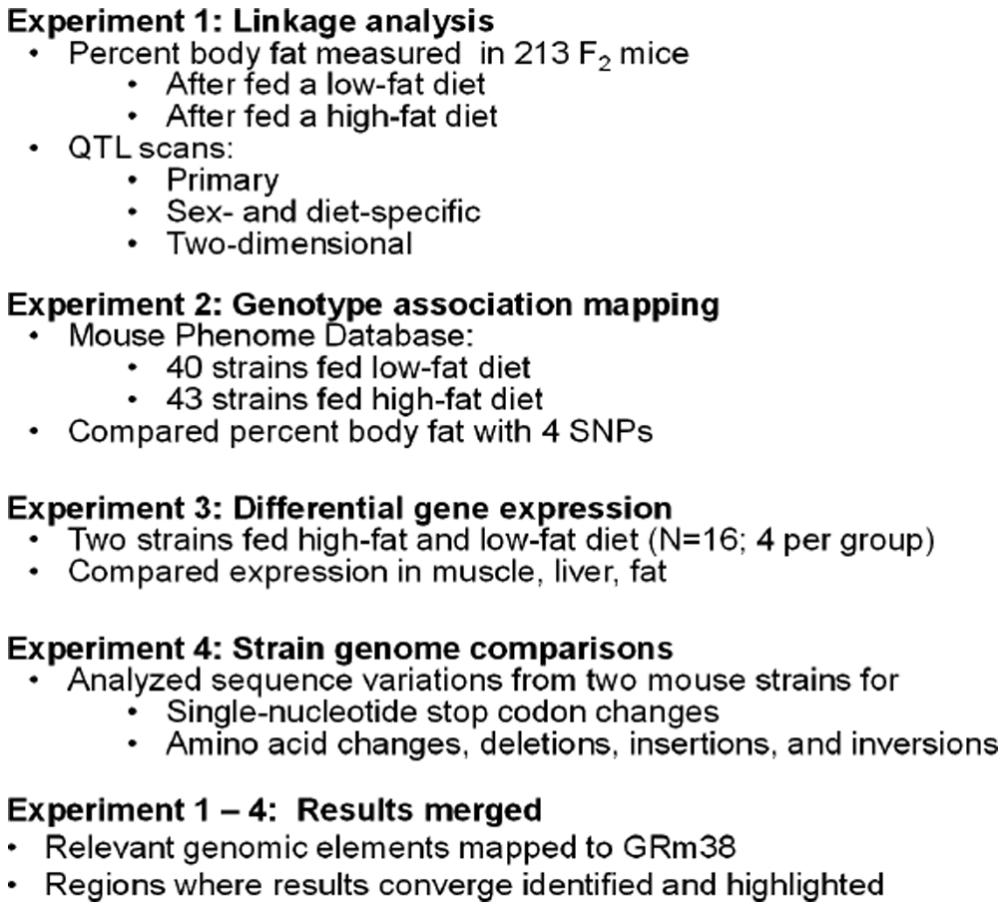
Experimental design. In Experiment 1, B6 x 129 F_2_ mice were fed a low- and then a high-fat diet. Body composition was measured at the end of each diet period, and QTL analyses were conducted for each diet condition. To narrow down a list of candidate genes that could account for the QTL, genotype association mapping (Experiment 2) was conducted using inbred strains and 4 million imputed SNPs. Differential gene expression analysis of tissues using microarrays (Experiment 3) indicated which genes in the QTL boundaries were differentially expressed between the parental B6 and 129 strains in liver, muscle, and adipose tissue. DNA genomic sequences from the QTL regions were compared between the parental strains (Experiment 4) to identify variants that might affect gene function. Results of all four experiments were compared to highlight genes identified by multiple methods.

Four experiments were conducted, a linkage analysis to identify regions of the genome associated with dietary obesity (Experiment 1), and three studies of the regions within the QTL boundaries: genotype association mapping (Experiment 2), differential gene expression analysis using microarrays (Experiment 3), and base-pair by base-pair strain genome comparisons of the DNA sequence for putative functional variants (Experiment 4). The goal was to filter the QTL regions for variants that were most likely to influence dietary obesity in mice. A chart of the experimental design is presented in **Figure 1**.

### Ethics Statement

The experiments involving animals were approved by the Institutional Care and Use Committee at the Monell Chemical Senses Center.

### Experiment 1, Linkage Analysis

#### Mice/diets

Females of the B6 inbred strain were obtained from the Jackson Laboratory (Bar Harbor, ME) and mated with two males from the incipient congenic strain 129P3/J.C57BL/6ByJ-*Tas1r3* (N_4_F_4_) to produce F_1_ and then F_2_ hybrid generations. This breeding strategy was undertaken to eliminate allelic variation of the sweet taste receptor gene (*Tas1r3*) in the 129 strain that markedly affects dietary behavior. Shortly after mice were born, the number of pups in each litter was recorded. Three-week-old litters were weaned, ear-marked, and grouped by sex, and the mice were housed in same-sex groups of 2–7 mice per cage until they were 18–20 weeks old, at which time they were individually housed for the duration of the experiment. In total, 213 F_2_ mice were bred and measured for body composition (described below). Before weaning, the litters and their parents were maintained on a “breeder” diet (Lab Diet 5015, PMI Nutrition International; 4.7 kcal/g, 11% fat, 70% carbohydrate, 19% protein). After weaning, mice were fed a regular chow (Teklad #8604, a low-energy diet; see **Table S1**). From 3 weeks to ∼6 months of age, mice were tested for their taste preferences as a part of a separate study. When these tests were completed, mice were fed a high-energy diet for 8 weeks.

#### Body composition

Percent body fat was measured twice, once at the end of each diet period. Measurements of fat and lean weight were made by dual energy X-ray absorptiometry (DEXA) using a Lunar PIXImus II densitometer (GE, software version 2.00; Lunar Corp., Madison, WI). This instrument was calibrated daily according to the manufacturer’s instructions using a quality control phantom (phantom values: percentage fat = 10.0%). Mice were anesthetized using a mixture of ketamine and xylazine and placed on a positioning tray ventral side down with the legs extended away from the body. Because some mice were longer than the imaging area (80×65 mm), the head of each mouse was excluded from analysis. The weight of body fat in grams was divided by total body weight and multiplied by 100 (percent body fat). Percent fat was used as the measure of obesity in this study because it is a common metric and hence facilitates comparison across studies. Prior work in our laboratory has validated the DEXA method using a range of mouse strains [4], and other investigators have used this method to conduct genetic analyses of body composition in mice (e.g., [27], [28], [29], [30]). We also included five additional phenotypes derived from these measures, described in **Table S2**.

#### Genotyping

Genomic DNA was extracted and purified from mouse tails by a sodium hydroxide method [31] or by proteinase K digestion followed by high-salt precipitation (Gentra/Qiagen, Valencia, CA). The F_2_ mice and the F_1_ and congenic progenitors were genotyped, as well as control DNA samples from the 129P3/J, C57BL/6ByJ, and C57BL/6J inbred strains. Genotyping was conducted in two steps, resulting in an average distance between markers of 12 Mb, with no gap greater than 40 Mb. First, simple sequence repeat markers known to be polymorphic between the parental strains were genotyped to evenly cover all 19 autosomes [32]. These fluorescently labeled microsatellite primers were amplified by PCR, and the products were scanned by an ABI 3100 capillary sequencer (Applied Biosystems, Forest City, CA). This genotyping was conducted by the Center for Inherited Disease Research (CIDR, Johns Hopkins University). Second, single-nucleotide polymorphisms (SNPs) were added to fill gaps (KBiosciences, Herts, UK). A few SNPs were also genotyped in our laboratory, using primers and fluorescently labeled probes designed to discriminate between alleles, using an ABI Prism 7000 real-time PCR system (ABI Assay-by-Design, Applied Biosystems, Foster City, CA). Genotypes were checked by determining whether they were compatible with the pre-existing haplotypes; suspicious genotypes such as those that created double recombinants were re-assayed. In addition, genotypes associated with coat and eye color were used as markers to verify the molecular genotypes, which were consistent with the expected haplotype in all cases. Markers and their physical positions on the chromosome based on build GRCm38 are listed in **Table S3**.

#### Statistical analysis overview

Linkage analysis was conducted using the algorithms implemented in R/qtl version 1.08–56 [33], in four successive steps: (a) a genetic map was estimated from the marker genotypes from the F_2_ mice, (b) covariates were identified using regression methods, (c) main effect QTLs and pairwise interactions were identified, and (d) the covariates and all main effect and interacting QTLs were incorporated into a model to determine how much phenotypic variance could be accounted for. Descriptive statistics and the initial assessment of covariate effects were conducted using Statistica 8.0 (StatSoft, Tulsa, OK).

#### Covariates

Litter size and age were used as covariates in all analyses because mice from larger litters had a lower percentage of body fat than did those from small litters (r = -0.31, p<0.05), and older mice had a higher percentage of body fat than did younger mice (r = 0.20, p<0.05).

#### Sex effects

Male mice are typically fatter than female mice, and QTLs for body composition are often influenced by sex (e.g., [34]). The QTLs may have similar effects on male and female mice (allowing for the increased fatness of male mice overall), or there may be an interaction between sex and genotype. To detect these interactions, we compared the LOD scores when sex was included as an additive covariate and the scores obtained when sex was included as both additive and interactive covariates. The difference between the LOD scores (ΔLOD) was used to determine whether a particular locus is influenced by sex, which we and others refer to as a “sex-dependent QTL” [35]. Specifically, we used a threshold of Δ2.0 so that our results could be directly compared with those of a similar study [36]. We applied a similar logic to detect diet-dependent QTL effects.

Genome-wide significant (*P*<0.05) and suggestive (*P*<0.63) thresholds were assessed from 1,000 permutations conducted for each one-dimensional scan with values (significant/suggestive) of 4.63/3.08 for the low-energy diet and 6.16/3.77 for the high-energy diet. For pairwise comparisons, we used a *P*<0.05 criterion, which yielded thresholds of 10.5 for the low-energy diet and 9.6 for the high-energy diet. We defined locus boundaries using confidence intervals computed using the “LODint” command in R/qtl using a 1-LOD drop as a criterion. In cases where inspection of the LOD score curves of a single chromosome suggested multiple QTLs, we included both peaks in a multiple regression model and evaluated the ΔLOD between the model with one QTL and the model with two QTLs. A second peak was accepted if the ΔLOD exceeded 2.0. In the full regression models, covariates, main effect QTLs, and interacting pairs were entered, and the contribution of each was evaluated. We assigned QTL names to loci (1) if they met the criterion for *significant* linkage or (2) if they met the criterion for *suggestive* linkage and also replicated suggestive linkages previously detected using similar parental strains, traits, and methodology. All QTL name assignments followed the guidelines established by the International Committee on Standardized Genetic Nomenclature for Mice.

### Experiment 2, Genotype Association Mapping

The goal of the genotype association mapping analysis was to find regions (within the locus boundaries) that are the most likely to contain the genetic variants that account for the observed QTL. Therefore, we extracted percent body fat phenotype data from the Mouse Phenome Database [37] from two studies: a 40-strain survey of 14- to 18-week-old mice fed a standard laboratory chow (low-energy diet; **Table S1**) using data originally collected in our laboratory [4], and a 43-strain survey of 15– to 17-week-old mice fed a high-energy diet for 8 weeks conducted at the Jackson Laboratory [38]. Percent body fat was measured in both strain surveys using the same method employed in our linkage analysis (Experiment 1).

We examined these data in conjunction with strain-specific nucleotide variants as described below. Data analysis was conducted using two statistical approaches as implemented in the program GEMMA [39], following the guidelines suggested by their developers and with imputation of 4 million SNPs genome-wide. Sex was used as a covariate in the analysis. Results were filtered by the locus boundaries, and the top 5% of nominally significant results (P<0.05) were extracted from each of the four QTL regions.

We also evaluated the genotype association mapping results in conjunction with the pattern of results from similar studies (i.e., multiple cross mapping), paying particular attention to the possibility that the causal variant might have arisen recently and thus would not be detected in other QTL mapping studies. Therefore, we extracted all previously published matching QTLs for comparison from the Mouse Genome Informatics database.

### Experiment 3, Differential gene expression

*Mice, diets, and tissues.* Four 6-week-old male C57BL/6J and four 6-week-old male 129S6/SvEvTac mice were fed a low-energy or a high-energy diet for 18 weeks (N = 16; 4 each group). Mice were euthanized and dissected; tissue was harvested from hind limb muscle, liver, and fat (gonadal adipose depot), and total RNA was isolated. These tissues were chosen because of their central role in energy output (muscle), storage (adipose tissue), and metabolism (liver). Methods of RNA preparation, hybridization (using Affymetrix mouse U74Av2 chips), and normalization were the same as those described previously [40].

Normalized data were extracted for genes within the locus boundaries suggested by the linkage results, and t-tests were performed to compare expression differences between the C57BL/6J and 129S6/SvEvTac strains. We extracted the top 5% of the most statistically significant results for each region for comparison with the results from Experiment 2 (genotype association mapping) and Experiment 4 (see below).

### Experiment 4, Strain Genome Comparisons

The genome sequence was analyzed for the 129P2/OlaHsd and C57BL/6J strains to evaluate potential candidate genes [41], [42], [43], [44]. These strains are closest in ancestry to the parental strains used in Experiment 1 that have fully sequenced genomes available [45]. Seven types of sequence variation were cataloged: (a) single-nucleotide changes that were predicted to result in the gain or loss of a stop codon, (b) single-nucleotide changes that were predicted to change an amino acid, (c) all single nucleotide polymorphisms, (d) deletions, (e) insertions (including transposable elements), (f) inversions and (g) copy number variants. The number and type of differences between the two strains from the locus boundaries were counted.

### Filtering of Candidate Genes

The results from the genotype association mapping (Experiment 2), differential gene expression analysis (Experiment 3), and strain genome comparisons (Experiment 4) were merged, and all relevant genomic elements (SNPs, microarray probes, genomic features such as stop codons) were mapped to GRCm38, the most recent map of the mouse genome available. We identified and highlighted regions within the locus boundaries where results converged, for example, a gene that differed in expression in one or more tissues (Experiment 3) and also contained a significant genotype-phenotype association when the panel of 43 inbred strains was evaluated (Experiment 2).

## Results

### Experiment 1, Linkage Analysis: Effects of the High-energy Diet

Before access to an energy-dense diet, male mice were about 25% body fat and females were about 21% body fat. After 8 weeks of eating an energy-dense diet, most mice were considerably fatter, with means of 38% body fat for males and 35% body fat for females (**Table 1**). Males were fatter than females [effect of sex: F(1,212) = 18.1 p<0.001, two-way ANOVA], and the high-energy diet led to an increase in percent body fat [effect of diet: F(1,212) = 782.24, p<0.001]. Male and female mice responded to the diet in a similar way [interaction between sex and diet: F(1,212) = 1.19, p = 0.28]. The fatness of individual mice before and after access to the high-energy diet was positively correlated (r = 0.49, p<0.001); that is, the fattest mice became fatter when the caloric density of their diet was increased.

**Table 1:** Characteristics of C57BL/6ByJ x 129P3/J F_2_ mice fed low- and high-energy diets (Experiment 1).

Trait or measure	Males	Females
N	111	102
Litter size	9±2 (4–13)	9±2 (4–13)
Age (range), months	8.0±0.3 (7.7–9.0)	8.0±0.4 (7.7–9.0)
Percent body fat, %		
Low-energy diet	25±5 (15–39)	21±5 (15–43)*
High-energy diet	38±5 (14–45)	35±10 (14–56)*

Values are mean±SD (minimum-maximum).

Age refers the age of the mouse at the end of the high-fat diet period.

* Male versus female, p<0.05.

#### Diet, sex, and allelic interactions

**Figure 2:**
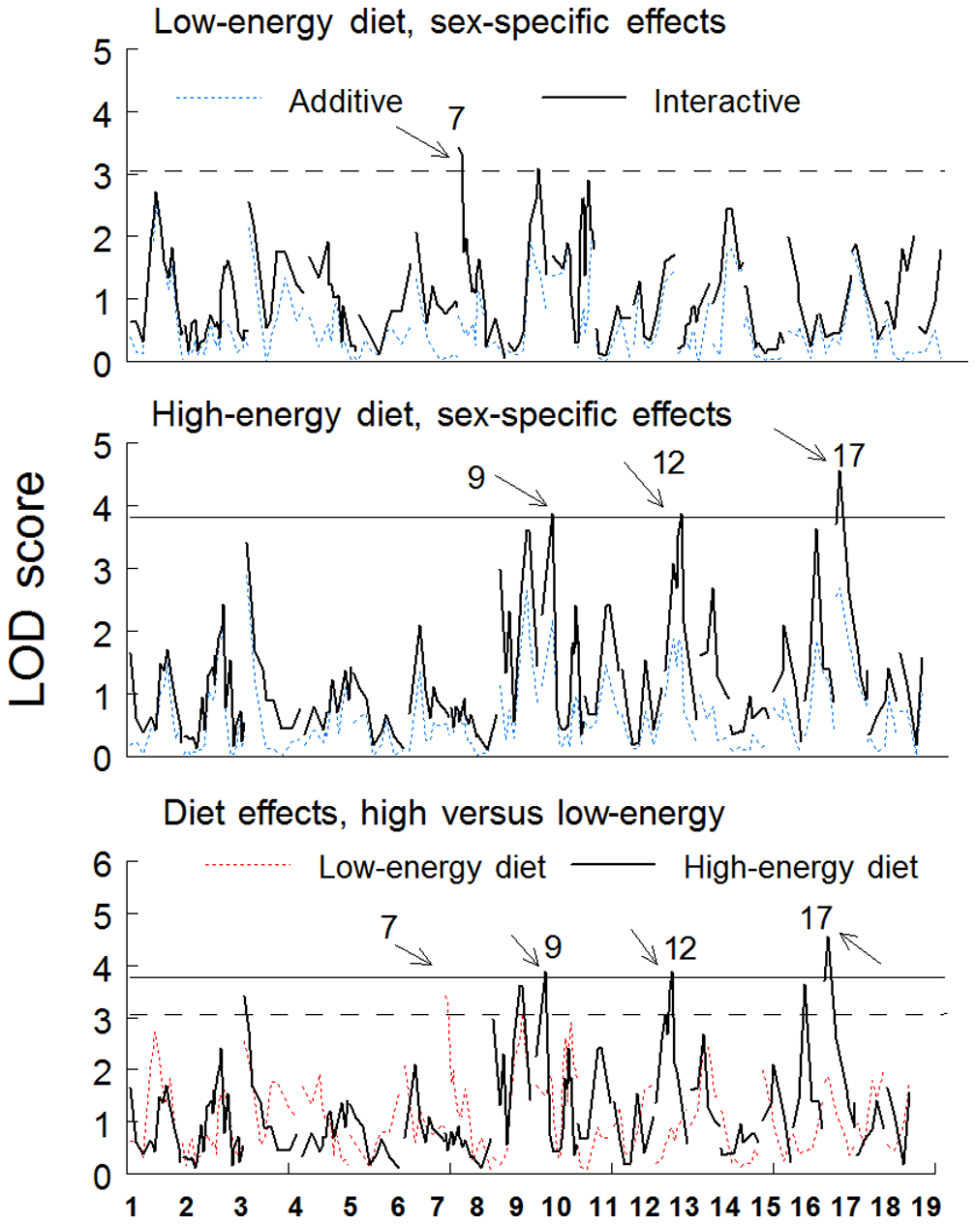
Genome-wide scans for the percentage of body fat (% body fat) of C57BL/6ByJ x 129P3/J F_2_ male and female mice fed low- and then high-energy diet (Experiment 1). Horizontal lines represent suggestive linkage thresholds determined by 1000 permutation tests [threshold = 3.08 when mice were fed the low-energy diet (dashed lines) and 3.77 when fed the high-energy diet (solid lines)]. (A and B) Sex-dependent QTLs are determined by the difference between the LOD scores when sex is used as an additive versus a combined additive and interactive covariate. Significant differences between the additive (dashed line) and additive plus interactive (solid line) models after the low-energy (A) and high-energy (B) diets are indicated by arrows. (C) Diet-dependent effects were determined by the difference in LOD scores between each diet period (arrows). The y-axis lists chromosome numbers. All analyses included age and litter size as covariates, and the diet-specific analysis in C was conducted using sex as an interactive covariate. Details about individual QTLs are shown in **Table 2**.

**Figure 3:**
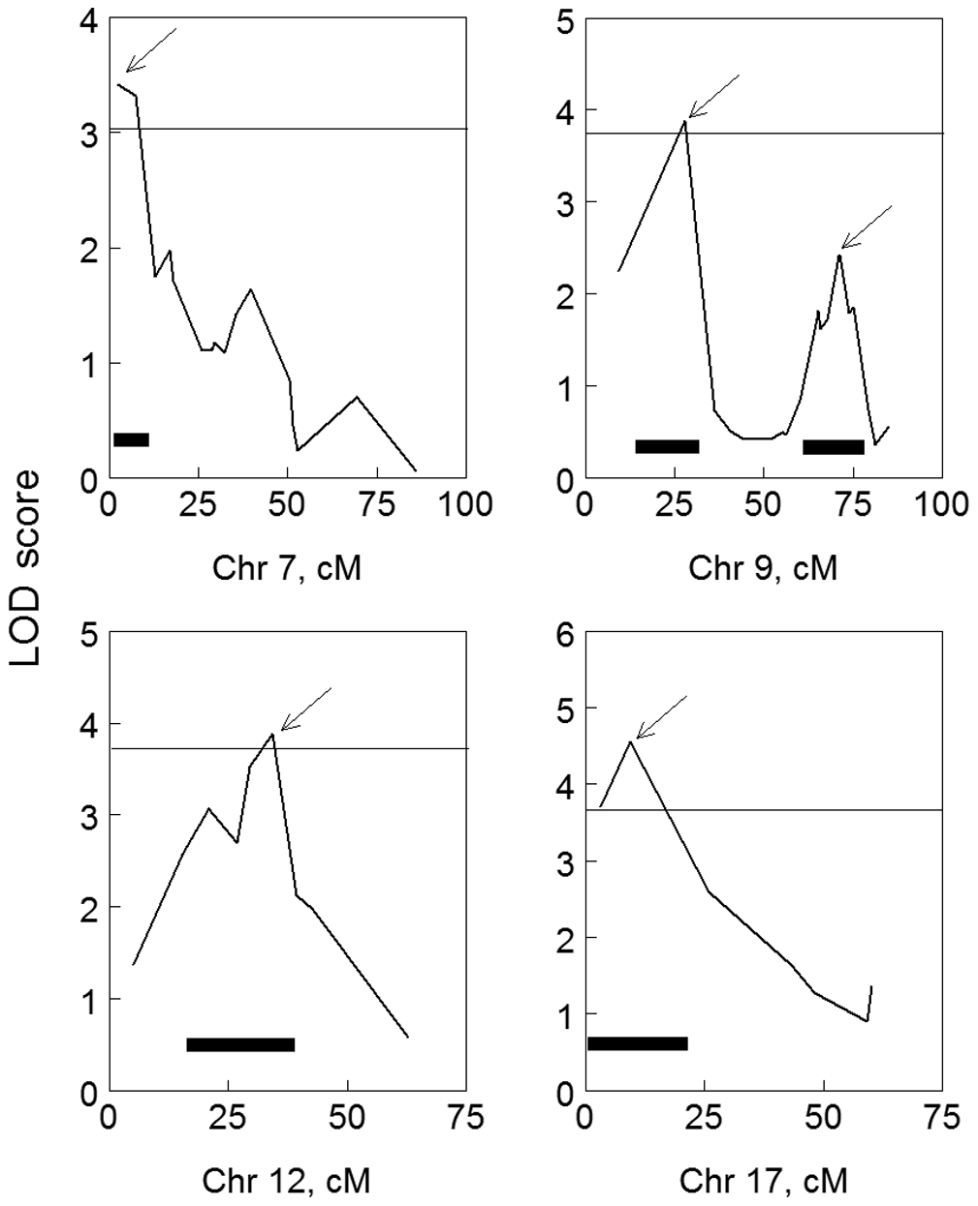
Interval maps of chr 7 (low-energy diet) and chr 9, 12, and 17 (high-energy diet), which each contained a QTL identified in the genome-wide scan linked to percent body fat in C57BL/6ByJ x 129P3/J F_2_ mice (Experiment 1). LOD score peaks are marked with arrows, and the associated locus boundaries are indicated by black bars below the peaks. Genetic locations (cM) are based on the experimental map from this genetic cross. Analysis of one- and two-QTL models indicated there are two peaks on chr 9. Significance thresholds are denoted by horizontal lines (for details, see **Figure 2**).

The genome scans identified four main effect QTLs on chr 7, 9, 12, and 17, all of which were diet dependent (**Figure 2**, **Table 2**). For chr 7, the QTL was associated with higher LOD scores when mice were fed the low-energy diet (*rs3687759*, 7.5 cM; ΔLOD = 2.88; **Figure 2A**). The other three QTLs were associated with higher LOD scores when mice were fed the high-energy diet (**Figure 2B**). Diet effects assessed using ΔLOD are indicated by arrows in **Figure 2C**: the locus on chr 9 (*D9Mit2*) had ΔLOD = 2.38; chr 12 (*rs3724069*), ΔLOD = 2.98; and chr 17 (*rs4231344*), ΔLOD = 2.68. The QTLs on chr 7 and 17 had clear single peaks (**Figure 3**); chr 9 and 12 appeared to have multiple peaks; there was statistical support for two peaks on chr 9 (Δ3.0 between models) but only one peak on chr 12 (Δ0.4). The second peak on chr 9 was located at 70.9 cM, and although it met the criterion for a distinct peak, it did not meet the criterion for suggestive linkage by itself. All QTLs were diet dependent.

**Table 2:** QTLs for percent body fat in C57BL/6ByJ×129P3/J F_2_ mice (Experiment 1).

Chr	Diet	LOD	Peak (cM)	Marker	Plus allele	Sex	ANOVA result
7	Low-energy	3.42	3	*D7Mit21*	129 females	Yes	Sex [F(1,198) = 21.5**]
					B6 males		Genotype [F(2,198) = 1.57]
							Sex by genotype [F(2,198) = 6.50**]
9	High-energy	3.88	28	*D9Mit2*	Overdominance^a^	No	Sex [F(1,195) = 11.69**]
					129		Genotype [F(2,195) = 5.64**]
							Sex by genotype [F(2,195) = 3.70*]
12	High-energy	3.88	34	*rs3724069*	129 females	Yes	Sex [F(1,205) = 7.97**]
							Genotype [F(2,205) = 4.59*]
							Sex by genotype [F(2, 205) = 4.79**]
17	High-energy	4.56	10	*rs4231344*	B6	No	Sex [F(1,198) = 7.22**]
							Genotype [F(2,198) = 5.97**]
							Sex by genotype [F(2,198) = 4.27*]

Chr = chromosome. cM = centimorgan based on the experimental map. Marker = nearest LOD score peak. “Plus” refers to the allele that increases the trait value. Sex = sex-dependent by the criterion described in the text. For percent variance, see **Table 4**. For locus boundaries, see **Table 5**.

a Overdominance means that phenotype of heterozygotes differs from phenotypes of both homozygotes. *p<0.05. **p<0.01.

**Figure 4:**
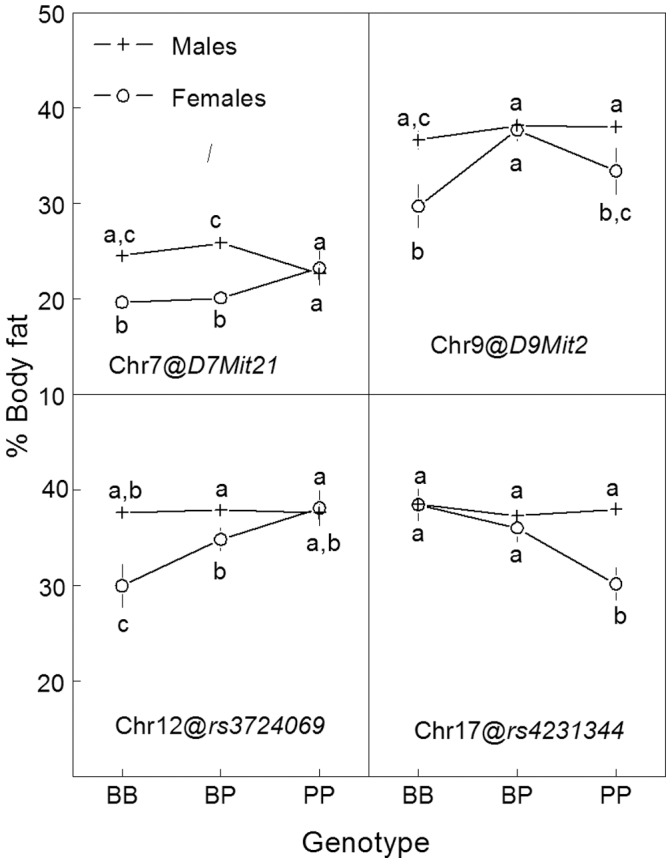
Percent body fat of F_2_ mice by sex and genotype (Experiment 1).

Two QTLs were sex dependent, on chr 7 and 12, ΔLOD = 2.72 and ΔLOD = 2.11, respectively (**Figure 2A,B**). For the sex-dependent linkage on chr 7, female mice homozygous for the allele derived from the 129 strain had higher body fat than did heterozygous mice and mice homozygous for the B6 strain (**Figure 4**, **Table 2**). For the sex-dependent QTL on chr 12, there was no effect of genotype on percent body fat in male mice, but there was an additive effect in female mice, with the 129 allele increasing body fat. Two other loci did not meet the ΔLOD criterion for sex-dependent linkage, i.e., on chr 9 and chr 17. For the QTL on chr 9, the mean of the heterozygous mice was higher than for either of the homozygous groups. For the QTL on chr 17, there was an additive effect, with the B6 allele increasing the percent body fat.

#### Pairwise interaction

**Figure 5:**
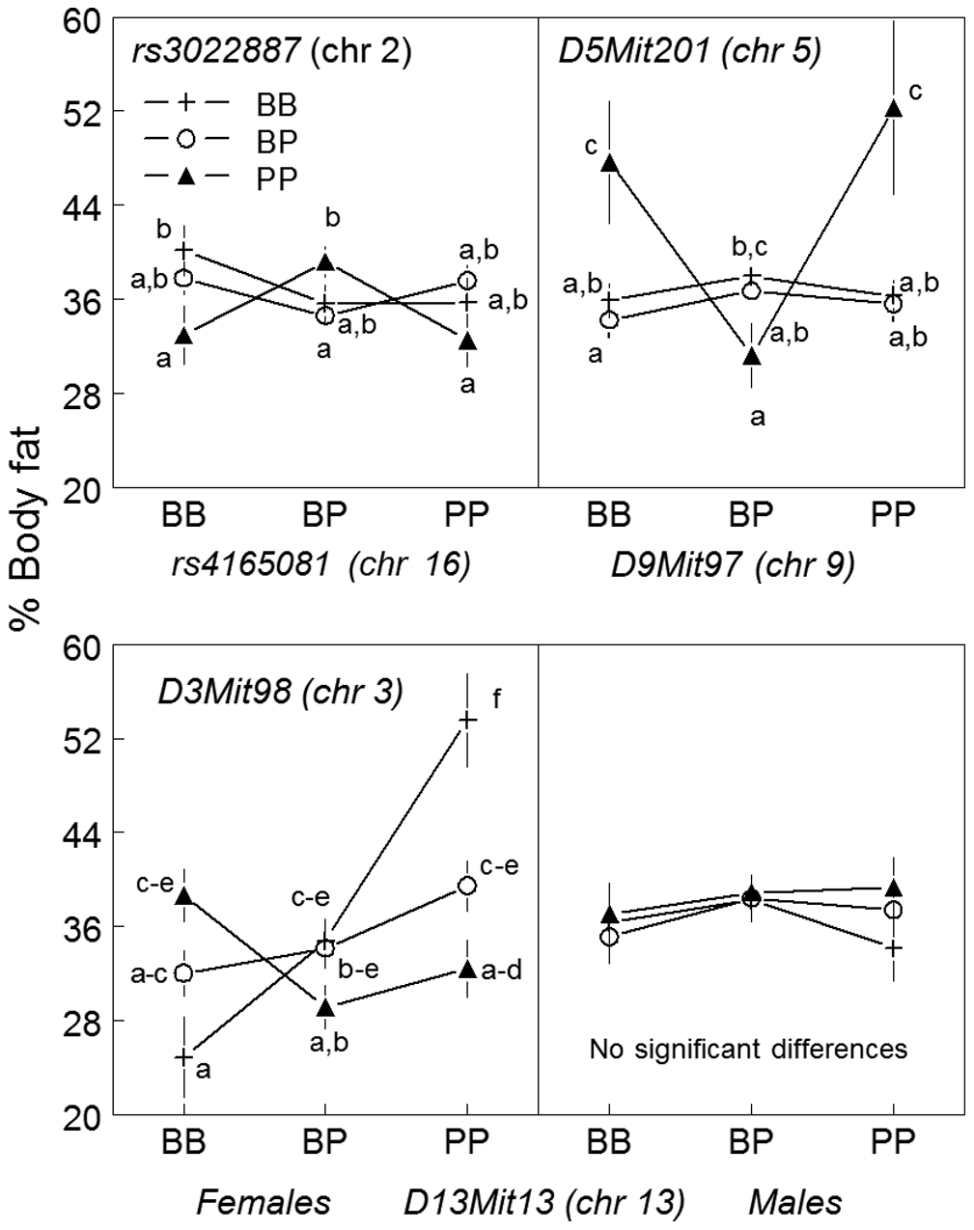
Percent body fat of F_2_ mice by genotype of interacting loci detected in the pairwise genome scan (Experiment 1): chr 2 and 16 (upper left), chr 5 and 9 (upper right), and chr 3 and 13 (females, lower left; males, lower right). The x-axis shows genotype: BB, homozygous for C57BL/6ByJ alleles; PP, homozygous 129P3/J strain alleles; BP, heterozygous. Values are mean ± SEM. Groups that do not share a common subscript (a-f) significantly differed by post hoc testing.

A pairwise genome scan failed to detect any interacting loci for percent body fat when mice ate a low-energy diet, but three pairs (chr 2×chr 16, chr 3×chr 13, and chr 5×chr 9) were detected when mice ate the high-energy diet (LOD = 9.7, 11.2, and 12.8, respectively). **Figure 5** shows mean percent body fat of mice grouped by genotype of the interacting markers and the results of post hoc testing. There was a sex by genotype interaction between loci on chr 3 and 13, and this interacting pair was sex dependent.

#### Modeling and percent variance

All main effect and pairwise QTLs, sex-dependent interactions found in the single-locus scan, and the covariates (sex, litter size, and age) were entered into a model, and their relative contribution to percent body fat on the low- or high-energy diet was assessed (**Table 3**). The amount of variance explained by genotype and other covariates nearly doubled when mice ate a high-fat compared with a low-fat diet (55.2% vs. 32.6%). This was due mostly to the larger number of main effect and pairwise QTLs for the high-fat feeding condition.

**Table 3:** Multiple regression analysis of variance for percent body fat in C57BL/6ByJ × 129P3/J F_2_ mice fed low- and high-energy diets (Experiment 1).

Type	Chr@Mb	df	% Var	F-value	P-value
Low-energy diet					
Sex		3	15.2	14.1	1.6x10^−8^
Litter size		1	5.5	15.4	8.6x10^−5^
Age		1	1.8	5.1	2.3x10^−2^
Main effect QTL	Chr 7@3.3	4	5.7	4.0	3.2x10^−3^
Sex^-^dependent QTL	Chr 7@3.3:Sex	2	4.4	6.2	1.9x10^−3^
Total			32.6		
High-energy diet					
Sex		3	3.9	4.0	2.9x10^−3^
Litter size		1	6.2	19.3	2.6x10^−6^
Age		1	0.2	0.6	0.40
Main effect QTLs	Chr2@69.1	6	0.8	0.4	0.87
	Chr3@86.2	6	1.8	0.9	0.38
	Chr5@75.2	6	7.3	3.7	2.9x10^−4^
	Chr9@37.4	2	1.0	1.5	0.17
	Chr9@50.7	6	8.3	4.3	6.9x10^−5^
	Chr12@78.2	4	2.6	2.0	5.2x10^−2^
	Chr13@56.5	6	2.2	1.1	0.25
	Chr16@19.9	6	4.8	2.4	9.8x10^−3^
	Chr17@23.7	2	2.8	4.2	7.0x10^−3^
Sex-dependent QTL	Chr12@78.2:Sex	2	1.1	1.7	0.13
Pairwise QTL	Chr3@86.2:Chr13@56.5	4	1.5	1.2	0.24
	Chr2@69.1:Chr16@19.9	4	3.4	2.6	1.6x10^−2^
	Chr5@75.2:Chr9@50.7	4	7.2	5.5	5.0x10^−5^
Total			55.2		

Chr@Mb = chromosome/megabase. % Var = percent variance accounted for. Interactions are denoted by a semicolon. Degrees of freedom (df) are predicated on main effects and interactions.

#### Other phenotypes

Additional linkage analyses were conducted for other phenotypes: body weight gain in grams (BwGG), body weight gain as a percent (BwGP), fat gain as a percent (FatGP), fat gain in grams (FatGG), and lean gain in grams (LeanGG). Several linkages (p<0.05) were located on chr 8 (FatGG and FatGP), chr 9 (LeanGG), chr 12 (BwGG and FatGG), and chr 15 (FatGP). These QTL regions overlap with previously described QTL and explain about 7–10% of the phenotypic variation in this mouse cross (**Table S2**).

#### Comparison with other studies

To further aid in the interpretation of these data, we whether QTLs from Experiment 1 matched those that were previously published. **Table 4** compares the locus boundaries, linkage peaks, and whether the dietary QTLs were detected in this and similar crosses. The results suggest that the QTLs on chr 9 and 17 are shared among many inbred strains and that the QTLs on chr 7 and 12 might be private to the parental stains used here – they have not been reported in other studies using similar methods.

**Table 4:** Comparison of QTLs from genome scans of B6 × 129 F_2_ mice fed high-energy diets (Experiment 1) with published data.

Chr	QTL name	C57BL/6J × 129S1/SvImJ		C57BL/6ByJ × 129P3/J		Same QTL?	Other mouse crosses	Reference
		Locus boundaries (Mb)	Location of LOD score peak (Mb)	Locus boundaries (Mb)	Location of LOD score peak (Mb)			
7	NA	50–100	88	0–26	4	No	No	NA
9	*Obq5*	0–40	30	8–50	37	Yes	KK × B6	[68]
12	*Obq34*	7–71	32	35–88	79	Yes	NZB × SM	[69]
17	NA	NA	NA	8–48	23	No	SM × NZB	[69], [70], [71], [72]
							B6 × 129	
							SM × NZO	
							C57L × AKR	
							B6 × A	

Chr = chromosome. C57BL/6J x 129S1/SvImJ refers to the results of a similar study that interbred these strains [36]. C57BL/6ByJ x 129P3/J refers to the results reported here. If the locus boundaries overlap, the QTLs are considered the same. Other crosses that have overlapping obesity QTLs are shown with the strain conferring the allele that increases the trait value first. NA = not applicable.

### Experiment 2, Genotype Association Mapping

**Figure 6:**
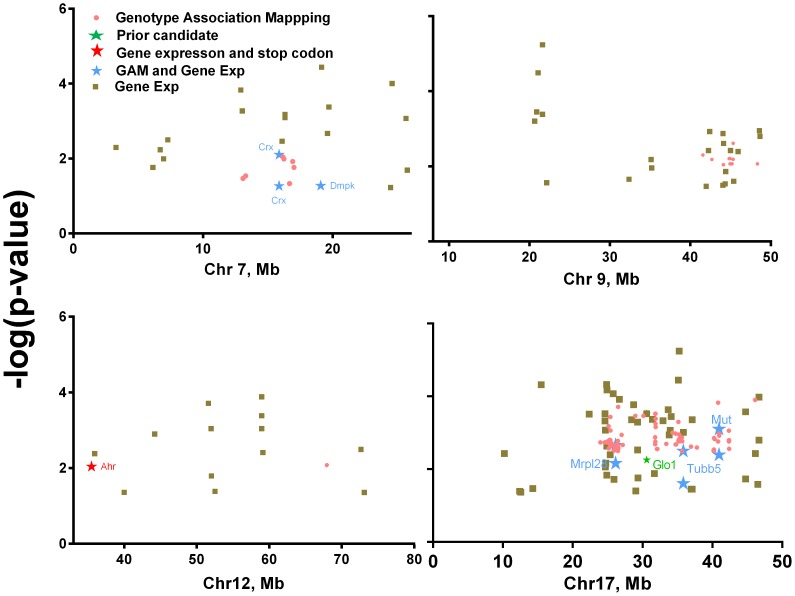
Results from genotype association mapping (Experiment 2), differential gene expression (Experiment 3), and strain genome comparison (Experiment 4) for the QTL boundaries on chr 7, 9, 12, and 17. The x-axis is Mb along the chromosome (markers mapped to GRCm38). Details of the top hits in the genotype association and differential gene expression analyses are listed in **Table S5**. Brown squares indicate results from the gene expression analysis (Gene Exp). Orange dots indicate results from the genotype association mapping (GAM). When gene expression and genotype association mapping results converged to a single location, the results are marked by blue stars. Some genes are marked twice by blue stars if they are differentially expressed in two tissues, e. g., adipose and muscle. When stop codons and gene expression converged to the same gene, the location is marked by red stars. The point of convergence of a stop codon and genotype association mapping results is marked by a purple star. *Glo1* is highlighted by a green star because it was previously nominated as a candidate gene in dietary obesity using similar methodologies.

The number of SNPs evaluated within each QTL boundary is as follows: chr 7, 3,045; chr 9, 11,069; chr 12, 10,836; and chr 17, 9,253. We then eliminated from consideration any SNP that was not allelic between the 129 and B6 strains, because we reasoned that it could not account for the QTLs identified in Experiment 1. We next culled the list to the top 5% of all the nominally significant results (p<0.05). We reasoned the exact p-values were less important than whether Experiments 2–4 converged to a single location within the QTL locus boundary. To that end, 219 SNPs were considered in the final analysis. These SNPs are reported in **Table S5** and are shown in **Figure 6** **.**

### Experiment 3, Differential Gene Expression

The goal of this analysis was to find genes within the QTL boundaries that were differentially expressed between the B6 and 129 strains. About 32% of the currently annotated genes (within the locus boundaries) were represented on this microarray. The p-values of individual genes were ranked and the top hits included in the list of candidate genes. The details of the filtering procedure are provided in **Table S4** and its caption. Thus the final list contained 103 genes, with 10 genes being differentially expressed in two or more tissues. These genes are listed in **Table S5** and are shown in **Figure 6** **.**

### Experiment 4, Strain Genome Comparisons

When the B6 and 129 genome sequences for the QTLs were cataloged, we found that each region (on chr 7, 9, 12, and 17) contained nucleotide changes that result in a gain or loss of a stop codon (**Table 5**). There were tens of thousands of single nucleotide polymorphisms (range 68,536 to 115,603), hundreds of changes that resulted in an amino acid substitution (coding SNPs) (range, 135–717); or a deletion (range, 264–422); and scores of insertions (range, 151–357), many of which were transposable elements. Many insertions or deletions were small (1 bp in length), but several were very large. There were at least two inversions within each locus boundary and three of the four QTL locus boundaries contained at least one copy number variations.

**Table 5:** B6 and 129 strain sequence variants within QTL boundaries (Experiment4).

Chr	Stopgain/loss	cSNPs	All SNPs	Deletions	Insertions	Inversions	CNV
7	11	717	68536	298	151	2	4
9	1	271	93160	264	260	3	1
12	2	135	97186	309	303	3	0
17	14	500	115603	422	357	4	9

Number of SNPs predicted to change amino acid sequence, 129P2/OlaHsd relative to C57BL/6J. cSNPs = coding SNPs: nucleotide changes predicted to result in an amino acid substitution. CNV = copy number variants.

#### Filtering of candidate genes

Results from Experiment 2 (genotype association) and Experiment 3 (differential gene expression) were mapped to their respective genomic locations (see **Figure 6** and **Table S5**). Differences in DNA sequence between the genomes were too numerous to be helpful because nearly every gene had some type of variant, so we focused on the highest quality sequence calls which predicted putative stop codons. When any two results from genotype, gene expression, and strain comparison stop codons converged to a single gene region, it was highlighted (**Figure 6**, stars). We also identified the *Glo1* gene because it has been implicated in dietary obesity as a candidate gene and was detected here, too, by differential gene expression analysis [46]. Seven genes were identified by these filtering methods, i.e., *Crx* and *Dmpk* (Chr 7), *Ahr* (Chr 12), *Mrpl28*, *Glo1*, *Tubb5*, and *Mut* (Chr 17). These analyses also indicated two regions of intense clustering of significant results, one short region of chr 9 from 41,561,916 to 48,343,282 and one longer region on chr 17 from 23,953,429 to 46,098,746. These dense clusters may represent hotspots for dietary obesity.

## Discussion

This study was designed to identify QTLs that contribute to the differences in dietary obesity between the B6 and 129 mouse strains. In this genome-wide search, we found four QTLs dependent on diet, two of which were also dependent on sex. These results highlight the importance of sex in the determination of dietary obesity in the mouse and suggest that the genetic pathways that increase fatness in male mice differ considerably from those in female mice [46]. The pattern of QTLs was also overwhelmingly determined by diet. One QTL was apparent only when mice were fed a low-energy diet, whereas the other three QTLs were apparent only when mice were fed a high-energy diet. Several pairwise interactions were also detected between loci, but only when mice were fed a high-fat diet. Extrapolating to humans, dietary differences among groups and cultures may be one reason for the lack of QTL replication from population to population. Part of the “missing heritability” of genome-wide association studies may arise because genotype associations are strongly tied to particular diets, but in humans intake is hard to quantify and difficult to control experimentally.

The pattern of the QTLs in this study was compared with those of other studies to see what could be learned about the probable origins of the underlying loci (Table 4). First, we considered the number and location of QTLs detected from a study using very similar parental strain pairing [36]. Two of the QTLs matched the ones detected here, which implies that the same ancestral allele accounts for both of these locations. The other two QTLs do not match. One of these–the QTL on chr 7–has not been detected in any other mouse cross, and thus we reasoned it may have arisen recently and be private to these substrains. New variants that arose after the creation of inbred mouse strains that affect body composition are common [47], [48].

The mice studied here were from the 129 and B6 strains, which are known to differ in their taste response to the main constituent of a high-energy diet used in dietary obesity studies, with the B6 strain preferring the taste quality of many high-energy diet (e.g., sweetness [49]). Some of the taste response to nutrients may be due to sensory differences between the strains, specifically alleles of the sweet receptor gene locus, which contribute to food preference. However, in this study we created an F_2_ population with only the B6 allele of this locus, which removed genetic variation known to affect sweet preference. Although the B6 strain is well-known for being susceptible to dietary obesity [50], the results of this linkage analysis suggest that the 129 allele increases fatness in the majority of the QTLs found in this cross. One explanation for this result is that the B6 strain is not susceptible to dietary obesity solely because of genomic variants. This suggestion arises because although all B6 mice are genetically identical, individual mice from within this strain differ markedly in weight gain when fed a high-energy diet [51]. It may be that the dietary obesity of the B6 mouse arises through epigenetic mechanisms [52], [53] that change how incoming fuel is oxidized and stored [54]. Dietary obesity may be due to a mix of genetic and epigenetic variation that affects taste preferences, food intake, and metabolism.

The loci highlighted by the candidate gene analysis were *Crx, Dmpk, Ahr, Mrpl28*, *Glo1*, *Tubb5*, and *Mut*. To gauge whether these genes have a plausible role in obesity, here we review what is already known about their function. The *Crx* gene on chr 7 is found in photoreceptors of the retina. The link to obesity comes from the observation that one group of genetic disorders in humans have both obesity and retinal degeneration as cardinal features (e.g., Bardet-Biedl syndrome). In addition, a genetically obese rodent strain with retinal degeneration has reduced *Crx* gene expression in the retina of obese versus lean strains of animals [55]. Exactly how a retinal protein affects obesity is unclear, but models of Bardet-Biedl syndrome suggest that ciliated sensory cells oversecrete peptides (e.g., insulin), which may explain the dual nature of the phenotype [56]. The *Dmpk* gene codes for a serine-theonine kinase found in muscle and when this gene is deleted in mice, it reduces insulin signaling in muscle tissue and increases adiposity [57], [58]. *Ahr* is a ligand-activated nuclear receptor transcription factor, and a previously published study suggests that congenic male mice with a donor region from the DBA strain are less susceptible to dietary obesity than are mice with an *Ahr* haplotype derived from host strain (B6) [59]. This study is hard to interpret for two reasons. The authors refer to the B6-derived haplotype as a high-activity allele but the genome sequencing indicates it contains a stop codon (*rs3021951*). Also, in the published study the genotype differences in dietary obesity are apparent in male mice (females were not studied) but in our data males did not differ by genotype. These two observations limit enthusiasm for this particular candidate gene. *Mrpl28* is a mitochondrial ribosomal protein that participates in oxidative phosphorylation [60], and therefore changes in expression of this gene might affect the ability to oxidize fat. *Glo1* has been nominated as a candidate gene for dietary obesity [12], [46] and was differentially expressed in fat and liver tissue in this study. *Tubb5* is a structural protein that is part of the microtubule system, and this particular protein is down-regulated in the plasma of mice fed a high-fat compared with low-fat diet [61]. The *Mut* gene produces *a* mitochondrial enzyme involved in the catabolism of amino acids and fatty acids, and humans with loss-of-function genetic variants secrete methylmalonic acid in their urine. This disorder is not associated with obesity in humans but might be associated with leanness, which has been overlooked clinically (Online Mendelian Inheritance in Man; #251000). Thus, all the genes highlighted have a plausible role in metabolism and obesity.

The use of the three methods to filter the QTL boundaries for candidate genes did yield plausible prospects, but these methods also have limitations. The main limitation for the genotype association mapping (Experiment 2) is that it is based on the assumption that the underlying genes that give rise to the QTL existed before the divergence of strains. Another limitation is that most inbred strains have not been phenotyped for their response to dietary obesity and therefore are currently unavailable for analysis, which limits its statistical power. The analysis of differential gene expression in three tissues between the parental strains for genes within the QTL boundaries (Experiment 3) had technical limitations because the number of genes on the microarray is much lower than the number of genes currently annotated. This is a problem that has largely been solved with RNA sequencing methods [62]. However, other problems with gene expression remain: the determination of the correct tissue and cell type to study for a particular QTL, and that genetic variation underpinning the QTL may not result in differences in gene expression. The genome comparison between the parental strains (Experiment 4) has the limitation that there are so many variants that investigators must make assumptions about which are more likely to be consequential. We chose to focus here on variants that create stop codons, but non-synonymous or intronic variants, or those within microRNAs [63] or non-coding RNAs [64], might be responsible for the phenotype. Lastly, it is plausible that several genes and their variants combine to create a QTL peak, and this is especially pertinent for obesity gene mapping because at least a third of all genes are involved in mouse body composition [65]. Thus, candidate gene analysis, while useful, does not replace the certainty of other approaches like the construction of congenic strains with the appropriate host and donor region. Such studies for the QTL identified on chr 9 are ongoing in our laboratory.

A feature of the design of Experiment 1 was that mice were fed a low-energy diet and then switched to a high-energy diet. Genotype-phenotype differences between these two periods are attributed to diet, although when mice were fed the high-energy diet they were also older than when they consumed the low-energy diet. This design was implemented to study gene × environment interaction using the same animals, but it has the disadvantage of confounding age and diet. Nevertheless it need not distract from the overall conclusions of the study – the mice were middle-aged by human standards, and this does have a strength in reflecting the type of obesity with aging that is often found in humans.

The amount of weight gained by animals when they are fed an energy-dense diet differs remarkably from one animal to the next, and in this study about 50% of the total variance could be explained by a mixture of factors, including sex and genotype. We confirm that the effect of genetic variation is highly context dependent [66]; in other words, the allelic effects differ greatly depending on whether the mouse is male or female or on which diet it has recently been fed. Considering that little variance is accounted for by genotype in human obesity studies, which total 10,000 times more subjects than in the animal study we report here [67], it is likely that human genotype effects on obesity are also highly context dependent and that the mixture of subjects with different diets dilutes the genetic effects, making them harder to detect.

## Supporting Information

Table S1 **Summary of diets used in linkage (Experiment 1), genotype association (Experiment 2), and expression (Experiment 3) analyses.**
(DOCX)

Table S2 **QTLs for additional phenotypes in C57BL/6ByJ X 129P3/J F_2_ (Experiment 1).**
(DOCX)

Table S3 **List of polymorphic markers genotyped and their physical map locations.**
(DOCX)

Table S4 **Filtering steps for gene expression with the QTL locus boundaries (Experiment 3).**
(DOCX)

Table S5 **Filtering of candidate genes.**
(DOCX)
