# Supplementary material for: QTL Analysis of Dietary Obesity in C57BL/6byj X 129P3/J F2 Mice: Diet- and Sex-Dependent Effects
Source: PLoS One. 2013 Jul 29;8(7):e68776. doi: 10.1371/journal.pone.0068776 (PMC3726688; doi:10.1371/journal.pone.0068776)
Supplement: Table S1 — Summary of diets used in linkage (Experiment 1), genotype association (Experiment 2), and expression (Experiment 3) analyses. (DOCX) [file pone.0068776.s001.docx]

**Table S1.** Summary of diets used in linkage (Experiment 1), genotype association (Experiment 2), and expression (Experiment 3) analyses

| Diet characteristics | Diet type | | | | | | |
| --- | --- | --- | --- | --- | --- | --- | --- |
|  | Low energy | | |  | High energy | | |
| Experiment | Linkage | Genotype | Expression |  | Linkage | Genotype | Expression |
| Vendor | Teklad | Dyets | Taconic |  | Dyets | Custom | Teklad |
| Name | #8604 | AIN-76A | NIH#31 |  | #113092 | Custom | TD.93075 |
| % Protein | 24 | 19 | 19 |  | 20 | 19 | 21 |
| % Carbohydrate | 72 | 69 | 76 |  | 40 | 47 | 24 |
| % Fat | 4 | 12 | 5 |  | 40 | 34 | 55 |
| kcal/g | 3.9 | 3.8 | 3.9 |  | 4.6 | 4.9 | 4.8 |

% protein, carbohydrate, and fat refer to percentage of total kilocalories. Kcal=kilocalories. “Custom” refers to a diet prepared by the investigator.
