# Supplementary material for: QTL Analysis of Dietary Obesity in C57BL/6byj X 129P3/J F2 Mice: Diet- and Sex-Dependent Effects
Source: PLoS One. 2013 Jul 29;8(7):e68776. doi: 10.1371/journal.pone.0068776 (PMC3726688; doi:10.1371/journal.pone.0068776)
Supplement: Table S2 — QTLs for additional phenotypes in C57BL/6ByJ X 129P3/J F2 (Experiment 1). (DOCX) [file pone.0068776.s002.docx]

**Table S2.** QTLs for additional phenotypes in C57BL/6ByJ X 129P3/J F_2_ (Experiment 1)

| Phenotype | Chr | Marker | LOD | Peak (cM) | Boundaries (cM) | % Var | Reference |
| --- | --- | --- | --- | --- | --- | --- | --- |
| BwGG | 12 | *rs3724069* | 3.76 | 34.3 | 15.3-39.4 | 7.3 | *Obq34 [*[*69*](#_ENREF_69)*]* |
| LeanGG | 9 | *rs4227916* | 3.92 | 70.9 | 67.8-79.2 | 7.4 | *Lbm7* [[73](#_ENREF_73)], *Lbm12* [[28](#_ENREF_28)]. |
| FatGG | 8 | *rs6237645* | 3.82 | 59.8 | 5.7-74.7 | 9.9 | *Obsty2* *[*[*15*](#_ENREF_15)*], Obq16 [*[*69*](#_ENREF_69)*,*[*70*](#_ENREF_70)*]* |
| FatGG | 12 | *rs3665793* | 3.74 | 20.9 | 5.1-39.4 | 9.7 | *Obq34 [*[*69*](#_ENREF_69)*]* |
| FatGP | 8 | *rs6237645* | 4.25 | 59.8 | 54.9-74.7 | 9.3 | *Obsty2* *[*[*15*](#_ENREF_15)*], Obq16[*[*69*](#_ENREF_69)*,*[*70*](#_ENREF_70)*]* |
| FatGP | 15 | *rs4230721* | 3.68 | 27.9 | 25.2-48.9 | 8.1 | *Dob3* [[74](#_ENREF_74)], *Dob4* [[75](#_ENREF_75)] |

For the linkage analysis, five additional phenotypes were created from the measures of fat and lean body composition obtained during the DEXA measures. The first DEXA measures were made after mice were fed a low-fat diet and then again after eating a high-fat diet. The two main traits in the main analysis were percent body fat measured after the low- and high-fat diet. The additional measures were body weight gain in grams (BwGG), lean weight gained in grams (LeanGG), and fat gained in grams (FatGG) between the two measurement periods. We also included a percent measure of change for fat weight [FatGP: (fat weight in grams after high-fat diet – fat weight in grams after the low-fat diet)/body weight after low fat diet)*100]. QTL analyses were conducted as described in the main methods.% var=percentage of phenotypic variance accounted for by each QTL. References are to previous studies that identified a QTL for dietary obesity. No QTLs were found for body weight gain as a percentage of starting weight, BwGP.
