# Supplementary material for: QTL Analysis of Dietary Obesity in C57BL/6byj X 129P3/J F2 Mice: Diet- and Sex-Dependent Effects
Source: PLoS One. 2013 Jul 29;8(7):e68776. doi: 10.1371/journal.pone.0068776 (PMC3726688; doi:10.1371/journal.pone.0068776)
Supplement: Table S3 — List of polymorphic markers genotyped and their physical map locations. (DOCX) [file pone.0068776.s003.docx]

**Table S3.** List of polymorphic markers genotyped and their physical map locations

| **Chr** | **Marker** | **bp** | **Chr** | **Marker** | **bp** | **Chr** | **Marker** | **bp** |
| --- | --- | --- | --- | --- | --- | --- | --- | --- |
| 1 | *D1Mit316* | 10380460 | 5 | *D5Mit387* | 28350370 | 10 | *D10Mit126* | 27014268 |
|  | *rs3666554* | 25674430 |  | *rs3023045* | 50574994 |  | *rs3696307* | 53743149 |
|  | *rs3022790* | 45308980 |  | *D5Mit201* | 75154707 |  | *rs3717445* | 82992866 |
|  | *D1Mit132* | 77146501 |  | *D5Mit314* | 109682321 |  | *D10Mit95* | 92502726 |
|  | *rs3678377* | 85791405 |  | *D5Mit95* | 124829257 |  | *D10Mit14* | 118661261 |
|  | *D1Mit60* | 93609154 |  | *D5Mit98* | 138218097 | 11 | *rs3023251* | 20953301 |
|  | *rs3716148* | 131290754 |  | *rs3722801* | 149795466 |  | *rs3696538* | 38862541 |
|  | *D1Mit102* | 147249521 | 6 | *D6Mit138* | 4503849 |  | *D11Mit86* | 54190116 |
|  | *D1Mit159* | 159661993 |  | *rs3024195* | 36614538 |  | *D11Mit4* | 68609258 |
|  | *rs4222821* | 169972291 |  | *rs3664697* | 50589762 |  | *D11Mit54* | 96285658 |
|  | *rs3678548* | 183916564 |  | *D6Mit209* | 75544357 |  | *D11Mit333* | 108713783 |
|  | *D1MIT17* | 189571266 |  | *D6Mit284* | 92607326 |  | *rs3675603* | 121255239 |
|  | *rs3699344* | 190444033 |  | *D6Mit36* | 104503361 | 12 | *D12Mit182* | 10870605 |
| 2 | *D2Mit1* | 3886090 |  | *rs3653718* | 136587259 |  | *D12Mit60* | 34789964 |
|  | *rs3698941* | 11043342 |  | *D6Mit198* | 139171278 |  | *rs3665793* | 46919106 |
|  | *rs3674936* | 20371737 | 7 | *D7Mit21* | 3266569 |  | *rs3691446* | 59816237 |
|  | *rs3681655* | 26368355 |  | *rs3687759* | 12942419 |  | *D12Mit91* | 71742864 |
|  | *rs3689602* | 30412836 |  | *rs3666902* | 26684250 |  | *rs3724069* | 78158653 |
|  | *rs3022883* | 37751250 |  | *rs3705780* | 35329734 |  | *rs3716095* | 87045535 |
|  | *D2Mit61* | 60690269 |  | *rs13479191* | 36132134 |  | *D12Mit118* | 91512220 |
|  | *rs3022887* | 69083134 |  | *D7Mit52* | 40072200 |  | *rs3682260* | 113264958 |
|  | *rs3699089* | 76108604 |  | *rs13479242* | 43564779 | 13 | *D13Mit16* | 20293462 |
|  | *rs3686727* | 100122595 |  | *rs3710949* | 44496336 |  | *D13Mit19* | 43767680 |
|  | *D2Mit100,* | 106537392 |  | *rs3719256* | 44538647 |  | *D13Mit13* | 56481437 |
|  | *rs3659207* | 109823371 |  | *D7Mit83* | 51798122 |  | *rs3717315* | 75877571 |
|  | *rs3681694* | 112284851 |  | *Oca2* | 56239760 |  | *D13Mit213* | 108247159 |
|  | *rs3659112* | 116084486 |  | *rs3710266* | 68526398 |  | *D13Mit151* | 115551794 |
|  | *D2Mit395* | 119524934 |  | *rs3713432* | 78097862 | 14 | *rs3689508* | 9760330 |
|  | *rs3712844* | 127499938 |  | *Tyr* | 87427405 |  | *D14Mit126* | 21873086 |
|  | *rs3726475* | 138571943 |  | *D7Mit323* | 100875961 |  | *rs3669686* | 33003239 |
|  | *rs3023694* | 144768591 |  | *rs3726275* | 107408577 |  | *D14Mit60* | 47097794 |
|  | *D2Mit285* | 152857303 |  | *rs13479461* | 111948552 |  | *D14Mit39* | 68548044 |
|  | *a* | 154791402 |  | *rs3658154* | 117504660 |  | *rs3693589* | 78038402 |
|  | *rs3693259* | 160967208 |  | *rs3678261* | 134089519 |  | *D14Mit106* | 100181106 |
|  | *rs3687512* | 166976257 |  | *rs4226997* | 144896765 |  | *D14Mit75* | 116913959 |
|  | *rs3689258* | 169487900 | 8 | *D8Mit155* | 4976622 |  | *rs3685710* | 123146197 |
|  | *D2Mit113* | 173354691 |  | *D8Mit289* | 28788000 | 15 | *D15Mit13* | 3460213 |
|  | *rs3708892* | 174861274 |  | *D8Mit190* | 35987886 |  | *rs3667271* | 42825752 |
|  | *rs3680965* | 180754894 |  | *rs3661085* | 54852681 |  | *rs4230721* | 54746207 |
| 3 | *rs3680834* | 11297853 |  | *D8Mit45* | 87305399 |  | *D15Mit107* | 84386498 |
|  | *D3Mit203* | 26936127 |  | *D8Mit47* | 106844170 |  | *D15Mit161.1* | 97011164 |
|  | *rs4223936* | 36449161 |  | *rs6237645* | 110017519 | 16 | *D16Mit107* | 5702276 |
|  | *rs3679479* | 59243784 |  | *rs3693295* | 122947693 |  | *rs4165081* | 19883079 |
|  | *D3Mit98* | 86181502 | 9 | *D9Mit250* | 8393644 |  | *rs4173519* | 36098517 |
|  | *D3Mit311* | 93017197 |  | *D9Mit2* | 37394902 |  | *D16Mit139* | 65669543 |
|  | *rs3712218* | 109769327 |  | *D9Mit97* | 50679793 |  | *D16Mit153* | 87583384 |
|  | *D3Mit256* | 136351594 |  | *rs30042362* | 57740083 |  | *rs4221067* | 95108047 |
|  | *rs4224279* | 144434506 |  | *rs30280752* | 61069253 | 17 | *D17Mit143.2* | 8443251 |
|  | *D3Mit19* | 147980947 |  | *rs3668451* | 79146160 |  | *rs4231344* | 23674962 |
| 4 | *D4Mit227* | 10000519 |  | *D9Mit198* | 91281997 |  | *D17Mit10* | 47534994 |
|  | *D4Mit196* | 39452128 |  | *rs4138352* | 94301623 |  | *D17Mit93* | 73800677 |
|  | *D4Mit17* | 63365314 |  | *rs3692530* | 98349029 |  | *D17Mit122* | 83054214 |
|  | *rs3722264* | 74030429 |  | *rs3679358* | 103229737 |  | *rs3711314* | 91788181 |
|  | *rs3726519* | 83820013 |  | *rs13480388* | 104113507 |  | *rs3023460* | 93199618 |
|  | *D4Mit9* | 95069362 |  | *rs13480395* | 105143604 | 18 | *D18Mit222* | 14587510 |
|  | *rs3664701* | 106219128 |  | *rs3023231* | 106888925 |  | *rs13483241* | 20152459 |
|  | *rs3719823* | 118395675 |  | *rs4227916* | 108052494 |  | *D18Mit194* | 43660828 |
|  | *D4Mit203* | 129572038 |  | *rs3685576* | 109059379 |  | *rs3023468* | 63484034 |
|  | *rs3681494* | 134357389 |  | *rs3679771* | 112866211 |  | *D18Mit186* | 72020438 |
|  | *rs3718220* | 143308886 |  | *rs3721068* | 113915010 |  | *rs3723904* | 84161774 |
|  | *rs4140148* | 150476349 |  | *D9Mit201* | 117436167 | 19 | *D19Mit78* | 7591433 |
|  | *D4Mit209* | 154415511 |  | *rs3694852* | 119670046 |  | *D19Mit96* | 21841620 |
|  | *Tas1r3* | 155859268 |  | *rs3706619* | 123642539 |  | *D19Mit88* | 37256935 |
| 5 | *D5Mit146* | 8840694 | 10 | *D10Mit213* | 20407440 |  | *D19Mit26* | 53057426 |

Marker positions were determined by BLAT searches against GRCm38 with primer/probe or flanking DNA sequences (see Electronic resources). The position of genes associated with coat color is integrated into this table: agouti (*a*), oculocutaneous albinism II (*Oca2*), and tyrosinase (*Tyr*). Markers starting with the letters ‘*rs*’ refer to single nucleotide polymorphisms (most genotyped by KBioscience), whereas those that begin with *D#Mit* are simple sequence repeats (genotyped by CIDR). bp=base pair. See text for other details.
