# Supplementary material for: QTL Analysis of Dietary Obesity in C57BL/6byj X 129P3/J F2 Mice: Diet- and Sex-Dependent Effects
Source: PLoS One. 2013 Jul 29;8(7):e68776. doi: 10.1371/journal.pone.0068776 (PMC3726688; doi:10.1371/journal.pone.0068776)
Supplement: Table S4 — Filtering steps for gene expression with the QTL locus boundaries (Experiment 3). (DOCX) [file pone.0068776.s004.docx]

**Table S4**. Filtering steps for gene expression with the QTL locus boundaries (Experiment 3)

| Filtering step | Chr 7 | Chr 9 | Chr 12 | Chr 17 | Totals |
| --- | --- | --- | --- | --- | --- |
| 1) Identify all genes within locus boundary | 766 | 609 | 493 | 1167 | 3035 |
| 2) Identify microarray probes within locus boundary | 175 | 196 | 178 | 435 | 984 |
| 3) Eliminate if p-value > 0.05 | 71 | 70 | 79 | 195 | 415 |
| 4) Top 5% of results | 26 | 29 | 27 | 65 | 148 |
| 5) Eliminate probes that map to multiple locations | 18 | 24 | 16 | 45 | 103 |
| 6) Other tissues included if p-value < 0.05 | 19 | 24 | 21 | 49 | 113 |

The table shows numbers of genes remaining after each filtering step of data analyses in Experiment 3.

a) All genes located within the QTL locus boundaries (shown in **Table 4**) were retrieved from Ensembl Genes 71 (GRCm38).

b) Probes from the microarray which fell within the locus boundaries were also retrieved and compared to the total possible number of genes.

c) P-values from each locus boundary for each tissue were pooled and ranked from smallest to largest.

d) The top five percent of genes with the lowest p-values were selected as candidates, provided they met a nominal threshold for inclusion (p<0.05).

e) Genes were further eliminated if the associated microarray probe mapped to more than one location in the genome.

f) If a particular gene-tissue combination remained on the list after these filtering steps, the other tissue results for that gene were re-included, provided the other tissue result was nominally significant. This step was undertaken to get a fuller picture of the profile of the candidate genes.
