# Supplementary material for: QTL Analysis of Dietary Obesity in C57BL/6byj X 129P3/J F2 Mice: Diet- and Sex-Dependent Effects
Source: PLoS One. 2013 Jul 29;8(7):e68776. doi: 10.1371/journal.pone.0068776 (PMC3726688; doi:10.1371/journal.pone.0068776)
Supplement: Table S5 — Filtering of candidate genes. (DOCX) [file pone.0068776.s005.docx]

**Table S5.** Filtering of candidate genes

| **Chr** | **bp** | **Identifier** | **Gene** | **-LOG(p-value)** | **Type** | **Tissue** |
| --- | --- | --- | --- | --- | --- | --- |
| 7 | 3289038 | 96285_at | Myadm | 2.30 | Microarray | liver |
| 7 | 6131489 | 161562_f_at | Zfp787 | 1.76 | Microarray | fat |
| 7 | 6703892 | 96765_at | Peg3 | 2.24 | Microarray | fat |
| 7 | 6961160 | 96433_at | Usp29 | 1.99 | Microarray | fat |
| 7 | 7282316 | 99672_at | Clcn4-2 | 2.50 | Microarray | liver |
| 7 | 12922290 | 99336_at | Rps5 | 3.83 | Microarray | muscle |
| 7 | 13032006 | 97841_at | Chmp2a | 3.27 | Microarray | muscle |
| 7 | 13086699 | rs31925874 | Vmn1r85 | 1.47 | GAM | NA |
| 7 | 13293789 | rs49403105 | Lig1 | 1.54 | GAM | NA |
| 7 | 15865947 | 99900_at | Crx | 2.10 | Microarray | fat |
| 7 | 15867119 | rs31900945 | Crx | 1.27 | GAM | NA |
| 7 | 16098458 | 98887_at | Napa | 2.46 | Microarray | liver |
| 7 | 16161378 | rs32234655 | Slc8a2 | 2.05 | GAM | NA |
| 7 | 16236095 | rs31328447 | Gpr77 | 1.99 | GAM | NA |
| 7 | 16327053 | 95703_at | Sae1 | 3.09 | Microarray | muscle |
| 7 | 16327053 | 95703_at | Sae1 | 3.18 | Microarray | liver |
| 7 | 16674487 | rs32089279 | Ceacam15 | 1.33 | GAM | NA |
| 7 | 16919523 | rs32483170 | Calm3 | 1.92 | GAM | NA |
| 7 | 17010273 | rs32122970 | Ppp5c | 1.76 | GAM | NA |
| 7 | 17013615 | rs31204953 | Ppp5c | 1.76 | GAM | NA |
| 7 | 17014977 | rs37550818 | Ppp5c | 1.76 | GAM | NA |
| 7 | 17015113 | rs31388840 | Ppp5c | 1.76 | GAM | NA |
| 7 | 18992092 | rs31285526 | Gm10676 | NA | Stop codon | NA |
| 7 | 19083849 | 93431_at | Dmpk | 1.28 | Microarray | fat |
| 7 | 19092454 | rs46709471 | Dmpk | NA | Stop codon | NA |
| 7 | 19149722 | 95049_at | Snrpd2 | 4.44 | Microarray | muscle |
| 7 | 19354431 | rs31796062 | Ercc1 | NA | Stop codon | NA |
| 7 | 19606217 | 103091_at | Relb | 2.67 | Microarray | liver |
| 7 | 19702805 | rs32422544 | Tomm40 | NA | Stop codon | NA |
| 7 | 19716644 | 99934_at | Pvrl2 | 3.38 | Microarray | liver |
| 7 | 19954192 | - | Rpl7a-ps8 | NA | Stop codon | NA |
| 7 | 19954493 | rs108120642 | Rpl7a-ps8 | NA | Stop codon | NA |
| 7 | 20844350 | - | Vmn1r115 | NA | Stop codon | NA |
| 7 | 24125144 | rs45936280 | Zfp112 | NA | Stop codon | NA |
| 7 | 24462500 | 102663_at | Plaur | 1.23 | Microarray | fat |
| 7 | 24587543 | 95660_at | Ethe1 | 4.00 | Microarray | muscle |
| 7 | 25155474 | rs217250090 | D930028M14Rik | NA | Stop codon | NA |
| 7 | 25155491 | rs239627027 | D930028M14Rik | NA | Stop codon | NA |
| 7 | 25659153 | 97249_at | Exosc5 | 3.07 | Microarray | liver |
| 7 | 25757273 | 99136_at | Axl | 1.69 | Microarray | fat |
| 9 | 20642878 | 94268_f_at | Ubl5 | 3.20 | Microarray | muscle |
| 9 | 20894352 | 96883_at | Eif3g | 3.45 | Microarray | muscle |
| 9 | 21067520 | 104044_at | Fdx1l | 4.49 | Microarray | muscle |
| 9 | 21616169 | 98463_at | Smarca4 | 3.39 | Microarray | liver |
| 9 | 21616169 | 98463_at |  | 5.24 | Microarray | muscle |
| 9 | 22126731 | 98859_at | Acp5 | 1.57 | Microarray | fat |
| 9 | 32372418 | 103972_at | Kcnj1 | 1.65 | Microarray | fat |
| 9 | 35124408 | 94067_at | Dcps | 2.18 | Microarray | liver |
| 9 | 35184551 | 103693_at | Tirap | 1.95 | Microarray | liver |
| 9 | 38595158 | rs248298255 | Olfr913 | NA | Stop codon | NA |
| 9 | 41561916 | rs33720673 |  | 2.30 | GAM | NA |
| 9 | 41964720 | 100888_at | Sorl1 | 1.46 | Microarray | fat |
| 9 | 42254177 | 102769_f_at | Sc5d | 2.42 | Microarray | liver |
| 9 | 42412316 | 103326_at | Tbcel | 2.92 | Microarray | liver |
| 9 | 42683964 | rs33692299 | Grik4 | 2.18 | GAM | NA |
| 9 | 44043384 | 99057_at | Thy1 | 1.50 | Microarray | fat |
| 9 | 44067021 | 92820_at | Usp2 | 2.87 | Microarray | muscle |
| 9 | 44097201 | rs32593954 | Mfrp | 2.04 | GAM | NA |
| 9 | 44125612 | rs32591353 | AC148328.1 | 2.04 | GAM | NA |
| 9 | 44134469 | 160458_at | Mcam | 2.61 | Microarray | liver |
| 9 | 44334715 | 93019_at | H2afx | 1.54 | Microarray | fat |
| 9 | 44403761 | 160361_at | Trappc4 | 1.86 | Microarray | liver |
| 9 | 44834041 | rs29925770 | Mll1 | 2.21 | GAM | NA |
| 9 | 44927513 | rs6401292 | Ube4a | 2.07 | GAM | NA |
| 9 | 44934204 | rs30080066 | Ube4a | 2.07 | GAM | NA |
| 9 | 44956234 | rs32640763 | Ube4a | 2.07 | GAM | NA |
| 9 | 44957201 | rs30521311 | Ube4a | 2.07 | GAM | NA |
| 9 | 44959810 | rs3700385 | Ube4a | 2.07 | GAM | NA |
| 9 | 44960384 | rs30137094 | Ube4a | 2.21 | GAM | NA |
| 9 | 44965076 | rs30425788 | Ube4a | 2.07 | GAM | NA |
| 9 | 44965462 | rs29832049 | Ube4a | 2.07 | GAM | NA |
| 9 | 44966528 | rs32641692 | Ube4a | 2.07 | GAM | NA |
| 9 | 44981793 | 92683_at | Cd3d | 2.42 | Microarray | fat |
| 9 | 45178286 | rs29935183 | Gm17099 | 2.07 | GAM | NA |
| 9 | 45332166 | rs30331044 | Tmprss13 | 2.61 | GAM | NA |
| 9 | 45332965 | rs50110293 | Tmprss13 | 2.19 | GAM | NA |
| 9 | 45403138 | 94827_at | Fxyd2 | 1.60 | Microarray | fat |
| 9 | 45937857 | 95023_at | Sidt2 | 2.39 | Microarray | liver |
| 9 | 48343282 | rs29594859 | Nxpe2 | 2.07 | GAM | NA |
| 9 | 48591877 | 101473_at | Nnmt | 2.95 | Microarray | liver |
| 9 | 48654311 | 92202_g_at | Zbtb16 | 2.80 | Microarray | muscle |
| 9 | 50603901 | 97477_at | Timm8b | 4.17 | Microarray | muscle |
| 9 | 50845301 | 103912_at | Ppp2r1b | 1.47 | Microarray | fat |
| 12 | 35497974 | 160495_at | Ahr | 2.04 | Microarray | liver |
| 12 | 35500692 | rs3021951 | Ahr | NA | Stop codon | NA |
| 12 | 35925886 | 100550_f_at | Cox6c | 2.38 | Microarray | liver |
| 12 | 40005447 | 92805_s_at | Arl4a | 1.35 | Microarray | fat |
| 12 | 44205897 | 96680_at | Dnajb9 | 2.90 | Microarray | muscle |
| 12 | 51608541 | 100878_at | Strn3 | 3.71 | Microarray | liver |
| 12 | 51988312 | 104243_r_at | 6530401N04Rik | 3.04 | Microarray | muscle |
| 12 | 52023003 | 94184_at | Gpr33 | 1.79 | Microarray | fat |
| 12 | 52516077 | 92247_at | Arhgap5 | 1.38 | Microarray | fat |
| 12 | 58958383 | 104709_at | Sec23a | 3.04 | Microarray | liver |
| 12 | 58958383 | 104709_at | Sec23a | 3.38 | Microarray | muscle |
| 12 | 58958383 | 93711_at | Sec23a | 3.88 | Microarray | muscle |
| 12 | 59129720 | 104033_at | Ctage5 | 2.41 | Microarray | liver |
| 12 | 67897276 | rs47355455 |  | 2.07 | GAM | NA |
| 12 | 67898308 | rs29165652 |  | 2.08 | GAM | NA |
| 12 | 67962368 | rs29131758 |  | 2.08 | GAM | NA |
| 12 | 67965691 | rs48561523 |  | 2.08 | GAM | NA |
| 12 | 67969259 | rs47236768 |  | 2.08 | GAM | NA |
| 12 | 67970266 | rs6165032 |  | 2.08 | GAM | NA |
| 12 | 67972981 | rs47856664 |  | 2.08 | GAM | NA |
| 12 | 67973245 | rs51685867 |  | 2.08 | GAM | NA |
| 12 | 67973886 | rs47965752 |  | 2.08 | GAM | NA |
| 12 | 67990154 | rs51180313 |  | 2.08 | GAM | NA |
| 12 | 72650356 | 95620_at | Dhrs7 | 2.49 | Microarray | liver |
| 12 | 73099609 | 93000_g_at | Six4 | 1.35 | Microarray | fat |
| 12 | 76066628 | rs29154989 | Syne2 | NA | Stop codon | NA |
| 12 | 80790532 | 96614_at | 4933426M11Rik | 3.39 | Microarray | muscle |
| 12 | 80953185 | 100341_g_at | Slc10a1 | 2.40 | Microarray | liver |
| 12 | 84754560 | 160344_at | Npc2 | 1.59 | Microarray | fat |
| 12 | 84754560 | 160344_at | Npc2 | 4.00 | Microarray | muscle |
| 12 | 84773270 | 96021_at | Isca2 | 3.13 | Microarray | muscle |
| 12 | 87147707 | 160350_at | Gstz1 | 2.99 | Microarray | liver |
| 12 | 87305058 | 100893_at | Sptlc2 | 1.17 | Microarray | fat |
| 17 | 10206471 | 160726_at | Qk | 2.43 | Microarray | liver |
| 17 | 12378609 | 161626_f_at | Plg | 1.39 | Microarray | fat |
| 17 | 12584189 | 102947_at | Slc22a2 | 1.37 | Microarray | fat |
| 17 | 14279506 | 96926_at | Smoc2 | 1.47 | Microarray | fat |
| 17 | 15475721 | 98113_at | Psmb1 | 4.32 | Microarray | muscle |
| 17 | 19065395 | rs33484325 | Vmn2r98 | NA | Stop codon | NA |
| 17 | 20279627 | rs29523845 | Vmn2r106 | NA | Stop codon | NA |
| 17 | 21129862 | rs243762129 | Gm7732 | NA | Stop codon | NA |
| 17 | 22361453 | 104694_at | Zfp758 | 3.51 | Microarray | liver |
| 17 | 23953429 | rs47366223 | Sbpl | 2.74 | GAM | NA |
| 17 | 23955010 | rs46652058 | Sbpl | 2.74 | GAM | NA |
| 17 | 23958393 | rs46875120 | Sbpl | 2.74 | GAM | NA |
| 17 | 24398536 | rs33292084 | Abca3 | NA | Stop codon | NA |
| 17 | 24570303 | rs50908848 | Pkd1 | 2.74 | GAM | NA |
| 17 | 24595937 | 97953_g_at | Tsc2 | 3.52 | Microarray | muscle |
| 17 | 24595937 | 97953_g_at | Tsc2 | 3.32 | Microarray | muscle |
| 17 | 24693191 | 160269_at | Gfer | 2.06 | Microarray | fat |
| 17 | 24693191 | 160269_at | Gfer | 3.07 | Microarray | liver |
| 17 | 24717370 | rs46815202 | Snhg9 | 2.74 | GAM | NA |
| 17 | 24736642 | 160275_at | Msrb1 | 2.16 | Microarray | fat |
| 17 | 24848896 | 99659_r_at | Fahd1 | 4.32 | Microarray | muscle |
| 17 | 24877107 | rs48774781 | Igfals | 2.75 | GAM | NA |
| 17 | 24878770 | 97987_at | Igfals | 1.83 | Microarray | fat |
| 17 | 24878770 | 97987_at | Igfals | 2.63 | Microarray | liver |
| 17 | 24895116 | 95730_at |  | 4.18 | Microarray | muscle |
| 17 | 25120553 | rs33473102 | Ptx4 | 3.18 | GAM | NA |
| 17 | 25143942 | rs29499788 | Clcn7 | 2.72 | GAM | NA |
| 17 | 25148561 | rs29502733 | Clcn7 | 2.72 | GAM | NA |
| 17 | 25154164 | rs29518622 | Clcn7 | 2.78 | GAM | NA |
| 17 | 25159255 | rs33777358 | Clcn7 | 2.72 | GAM | NA |
| 17 | 25159369 | rs33777357 | Clcn7 | 2.72 | GAM | NA |
| 17 | 25159567 | rs33777356 | Clcn7 | 2.72 | GAM | NA |
| 17 | 25159967 | rs33777354 | Clcn7 | 2.72 | GAM | NA |
| 17 | 25161851 | rs33775171 | Clcn7 | 2.72 | GAM | NA |
| 17 | 25161893 | rs33775170 | Clcn7 | 2.72 | GAM | NA |
| 17 | 25162663 | rs33508968 | Clcn7 | 2.72 | GAM | NA |
| 17 | 25162753 | rs29521732 | Clcn7 | 2.72 | GAM | NA |
| 17 | 25165927 | rs33495454 | Clcn7 | 2.72 | GAM | NA |
| 17 | 25167871 | rs33781390 | Ccdc154 | 2.72 | GAM | NA |
| 17 | 25168090 | rs33147411 | Ccdc154 | 2.57 | GAM | NA |
| 17 | 25290973 | rs33689023 |  | 2.81 | GAM | NA |
| 17 | 25366550 | 93622_at | Tpsb2 | 2.40 | Microarray | liver |
| 17 | 25387976 | rs33775031 | Cacna1h | 2.79 | GAM | NA |
| 17 | 25396404 | rs33177954 | Cacna1h | 2.62 | GAM | NA |
| 17 | 25397305 | rs33779025 | Cacna1h | 2.67 | GAM | NA |
| 17 | 25400645 | rs33777241 | Cacna1h | 3.43 | GAM | NA |
| 17 | 25401004 | rs33777236 | Cacna1h | 3.06 | GAM | NA |
| 17 | 25401370 | rs33776162 | Cacna1h | 2.62 | GAM | NA |
| 17 | 25401571 | rs33776160 | Cacna1h | 3.43 | GAM | NA |
| 17 | 25402103 | rs33685613 | Cacna1h | 2.62 | GAM | NA |
| 17 | 25402361 | rs33144741 | Cacna1h | 3.43 | GAM | NA |
| 17 | 25403283 | rs33774221 | Cacna1h | 2.62 | GAM | NA |
| 17 | 25477653 | rs33423035 | Tekt4 | 2.70 | GAM | NA |
| 17 | 25532849 | rs33563315 |  | 2.70 | GAM | NA |
| 17 | 25537028 | rs29499525 |  | 2.70 | GAM | NA |
| 17 | 25537115 | rs33099262 |  | 2.70 | GAM | NA |
| 17 | 25790508 | 94933_at | Fam173a | 4.07 | Microarray | muscle |
| 17 | 25875464 | 98124_at | 0610011F06Rik | 1.72 | Microarray | fat |
| 17 | 25887438 | rs51692858 | Rab40c | 2.51 | GAM | NA |
| 17 | 26078905 | rs29524058 | Decr2 | 2.66 | GAM | NA |
| 17 | 26080833 | rs33634271 | Decr2 | 2.66 | GAM | NA |
| 17 | 26081590 | rs52093512 | Decr2 | 2.62 | GAM | NA |
| 17 | 26082045 | rs46301286 | Decr2 | 2.66 | GAM | NA |
| 17 | 26082671 | rs51459547 | Decr2 | 2.60 | GAM | NA |
| 17 | 26083791 | rs51116003 | Decr2 | 2.60 | GAM | NA |
| 17 | 26087860 | rs47002262 | Nme4 | 2.66 | GAM | NA |
| 17 | 26088567 | rs33516151 | Nme4 | 2.66 | GAM | NA |
| 17 | 26090748 | rs33484700 | Nme4 | 2.66 | GAM | NA |
| 17 | 26091071 | rs33679207 | Nme4 | 2.66 | GAM | NA |
| 17 | 26091324 | rs33553459 | Nme4 | 2.60 | GAM | NA |
| 17 | 26110500 | rs49944470 | Tmem8 | 2.60 | GAM | NA |
| 17 | 26110708 | rs6182550 | Tmem8 | 2.66 | GAM | NA |
| 17 | 26110802 | rs33643592 | Tmem8 | 2.60 | GAM | NA |
| 17 | 26120560 | rs50411167 | Mrpl28 | 2.66 | GAM | NA |
| 17 | 26120608 | rs33211561 | Mrpl28 | 2.66 | GAM | NA |
| 17 | 26123520 | 96652_at | Mrpl28 | 2.16 | Microarray | liver |
| 17 | 26126830 | rs49451603 | Mrpl28 | 2.60 | GAM | NA |
| 17 | 26127259 | rs50041747 | Mrpl28 | 2.66 | GAM | NA |
| 17 | 26127830 | rs33498885 | Mrpl28 | 2.60 | GAM | NA |
| 17 | 26129587 | rs33719068 | Mrpl28 | 2.60 | GAM | NA |
| 17 | 26129867 | rs50304848 | Mrpl28 | 2.60 | GAM | NA |
| 17 | 26131689 | rs29539223 |  | 2.60 | GAM | NA |
| 17 | 26145326 | rs46737655 | Axin1 | 2.60 | GAM | NA |
| 17 | 26145371 | rs51491423 | Axin1 | 2.66 | GAM | NA |
| 17 | 26147265 | rs33180709 | Axin1 | 2.60 | GAM | NA |
| 17 | 26149686 | rs33356646 | Axin1 | 2.66 | GAM | NA |
| 17 | 26149859 | rs33599882 | Axin1 | 2.66 | GAM | NA |
| 17 | 26150015 | rs45825959 | Axin1 | 2.66 | GAM | NA |
| 17 | 26153934 | rs48368915 | Axin1 | 2.66 | GAM | NA |
| 17 | 26181116 | rs45991042 | Axin1 | 2.66 | GAM | NA |
| 17 | 26182720 | rs33617504 | Axin1 | 2.60 | GAM | NA |
| 17 | 26182997 | rs33087660 | Axin1 | 2.60 | GAM | NA |
| 17 | 26183433 | rs33648026 | Axin1 | 2.66 | GAM | NA |
| 17 | 26183519 | rs33684371 | Axin1 | 2.66 | GAM | NA |
| 17 | 26183640 | rs29502157 | Axin1 | 2.66 | GAM | NA |
| 17 | 26185983 | rs4136360 | Axin1 | 2.66 | GAM | NA |
| 17 | 26188497 | rs46090670 | Axin1 | 2.66 | GAM | NA |
| 17 | 26188632 | rs33716601 | Axin1 | 2.60 | GAM | NA |
| 17 | 26189012 | rs48731442 | Axin1 | 2.66 | GAM | NA |
| 17 | 26189188 | rs33464239 | Axin1 | 2.60 | GAM | NA |
| 17 | 26190263 | rs47133774 | Axin1 | 2.60 | GAM | NA |
| 17 | 26190985 | rs29519720 | Axin1 | 2.66 | GAM | NA |
| 17 | 26191166 | rs33675999 | Axin1 | 2.66 | GAM | NA |
| 17 | 26192057 | rs33565718 | Axin1 | 2.60 | GAM | NA |
| 17 | 26192076 | rs48377532 | Axin1 | 2.66 | GAM | NA |
| 17 | 26193324 | rs33049426 | Axin1 | 2.60 | GAM | NA |
| 17 | 26193779 | rs46615715 | Axin1 | 2.66 | GAM | NA |
| 17 | 26194143 | rs47565786 | Axin1 | 2.60 | GAM | NA |
| 17 | 26194174 | rs33383246 | Axin1 | 2.60 | GAM | NA |
| 17 | 26194888 | rs33135076 | Axin1 | 2.66 | GAM | NA |
| 17 | 26205638 | rs46884122 | Rgs11 | 2.66 | GAM | NA |
| 17 | 26207580 | rs33632109 | Rgs11 | 2.66 | GAM | NA |
| 17 | 26350411 | rs47157021 |  | 2.68 | GAM | NA |
| 17 | 26452130 | rs48301479 |  | 2.55 | GAM | NA |
| 17 | 26453362 | rs51511546 |  | 2.52 | GAM | NA |
| 17 | 26458428 | rs47978323 |  | 2.55 | GAM | NA |
| 17 | 26458909 | rs46883246 |  | 2.55 | GAM | NA |
| 17 | 26461089 | rs29503376 |  | 2.55 | GAM | NA |
| 17 | 26476604 | rs33102053 |  | 2.77 | GAM | NA |
| 17 | 26480405 | rs33052434 |  | 2.50 | GAM | NA |
| 17 | 26480614 | rs33694723 |  | 2.50 | GAM | NA |
| 17 | 26482187 | rs50642436 |  | 2.55 | GAM | NA |
| 17 | 26484097 | rs33441588 |  | 2.55 | GAM | NA |
| 17 | 26485551 | rs33249665 |  | 2.55 | GAM | NA |
| 17 | 26501798 | rs33531579 | Dusp1 | 3.71 | GAM | NA |
| 17 | 26513331 | rs33315495 | Dusp1 | 2.56 | GAM | NA |
| 17 | 26524798 | rs33675435 |  | 2.60 | GAM | NA |
| 17 | 26676396 | 94301_at | Atp6v0e | 3.91 | Microarray | muscle |
| 17 | 26996567 | rs29518468 | Ggnbp1 | 2.94 | GAM | NA |
| 17 | 27141204 | rs29505887 | Ip6k3 | 2.66 | GAM | NA |
| 17 | 28399095 | 94297_at | Fkbp5 | 3.36 | Microarray | muscle |
| 17 | 28691342 | 99978_s_at | Mapk14 | 3.77 | Microarray | muscle |
| 17 | 28958011 | rs33283793 | Kctd20 | 3.46 | GAM | NA |
| 17 | 29032673 | 101003_at | Srsf3 | 1.40 | Microarray | fat |
| 17 | 29268788 | 95517_i_at | BC004004 | 2.53 | Microarray | liver |
| 17 | 29268788 | 95517_i_at | BC004004 | 3.29 | Microarray | muscle |
| 17 | 29332072 | 95689_at | Mtch1 | 1.76 | Microarray | fat |
| 17 | 30107582 | rs33804897 | Zfand3 | 3.46 | GAM | NA |
| 17 | 30592866 | 93269_at | Glo1 | 2.25 | Microarray | fat |
| 17 | 30592866 | 93269_at | Glo1 | 3.52 | Microarray | liver |
| 17 | 31386234 | 102338_at | Pde9a | 3.37 | Microarray | liver |
| 17 | 31677933 | 102393_at | Cryaa | 1.88 | Microarray | fat |
| 17 | 31798481 | rs51095703 |  | 2.68 | GAM | NA |
| 17 | 31803237 | rs33296436 |  | 2.88 | GAM | NA |
| 17 | 31808824 | rs47248579 |  | 3.52 | GAM | NA |
| 17 | 31809153 | rs51035127 |  | 2.75 | GAM | NA |
| 17 | 31810594 | rs47853634 |  | 3.52 | GAM | NA |
| 17 | 31812245 | rs50572917 |  | 3.52 | GAM | NA |
| 17 | 31813475 | rs33515914 |  | 3.52 | GAM | NA |
| 17 | 31839389 | rs29502583 | Sik1 | 3.25 | GAM | NA |
| 17 | 31840554 | rs33343744 | Sik1 | 3.52 | GAM | NA |
| 17 | 31856886 | rs33078361 | Sik1 | 3.16 | GAM | NA |
| 17 | 31879760 | rs49555973 |  | 3.38 | GAM | NA |
| 17 | 31879900 | rs47099984 |  | 3.38 | GAM | NA |
| 17 | 32064860 | rs49206246 | Rrp1b | 2.55 | GAM | NA |
| 17 | 32108340 | rs48688395 |  | 2.55 | GAM | NA |
| 17 | 32924688 | 104129_at | Cyp4f13 | 3.33 | Microarray | liver |
| 17 | 33404347 | rs33232387 |  | 2.69 | GAM | NA |
| 17 | 33646233 | 96266_at | Hnrnpm | 3.63 | Microarray | muscle |
| 17 | 33810520 | 96785_at | Kank3 | 2.94 | Microarray | liver |
| 17 | 33938821 | 95158_at | H2-Ke2 | 3.07 | Microarray | liver |
| 17 | 34092340 | 102274_at | H2-Oa | 3.44 | Microarray | muscle |
| 17 | 34291932 | rs51246139 | H2-Aa | 3.00 | GAM | NA |
| 17 | 34735640 | rs45727786 | C4b | 2.68 | GAM | NA |
| 17 | 34735764 | rs13475139 | C4b | 2.68 | GAM | NA |
| 17 | 34739451 | rs33435966 | C4b | 2.68 | GAM | NA |
| 17 | 34740261 | rs33413496 | C4b | 2.68 | GAM | NA |
| 17 | 34742710 | rs48296365 | Stk19-ps1 | 2.97 | GAM | NA |
| 17 | 34808069 | rs8247250 | Cyp21a1 | 2.68 | GAM | NA |
| 17 | 34808354 | rs45899455 | C4a | 2.68 | GAM | NA |
| 17 | 35111026 | rs49645509 | Ly6g5b | 2.78 | GAM | NA |
| 17 | 35111116 | rs50821011 | Ly6g5b | 2.78 | GAM | NA |
| 17 | 35114074 | rs33467758 | Ly6g5b | 3.30 | GAM | NA |
| 17 | 35128997 | 93840_at | Apom | 4.45 | Microarray | liver |
| 17 | 35206092 | rs47855470 | Lta | 2.78 | GAM | NA |
| 17 | 35227103 | rs48733829 | Nfkbil1 | 2.78 | GAM | NA |
| 17 | 35230039 | rs50424609 | Atp6v1g2 | 2.95 | GAM | NA |
| 17 | 35233002 | rs33334663 | Atp6v1g2 | 2.95 | GAM | NA |
| 17 | 35237958 | rs33128146 | SNORD83 | 2.83 | GAM | NA |
| 17 | 35241746 | 160384_at | Ddx39b | 5.24 | Microarray | muscle |
| 17 | 35250397 | rs49912883 | SNORD83 | 2.78 | GAM | NA |
| 17 | 35250601 | rs51821957 | SNORD83 | 2.77 | GAM | NA |
| 17 | 35613906 | rs46240455 | Gm9573 | 2.87 | GAM | NA |
| 17 | 35636907 | rs46463169 | Sfta2 | 2.76 | GAM | NA |
| 17 | 35637583 | rs48027729 | Dpcr1 | 2.76 | GAM | NA |
| 17 | 35641842 | rs33429320 | Sfta2 | 2.76 | GAM | NA |
| 17 | 35643041 | rs33166555 | Sfta2 | 2.50 | GAM | NA |
| 17 | 35643994 | rs29523802 | Sfta2 | 2.76 | GAM | NA |
| 17 | 35668511 | rs47061363 | Gtf2h4 | 2.76 | GAM | NA |
| 17 | 35833926 | 94788_f_at | Tubb5 | 1.61 | Microarray | fat |
| 17 | 35833926 | 94789_r_at |  | 3.01 | Microarray | liver |
| 17 | 35834134 | rs3682923 | Tubb5 | 2.50 | GAM | NA |
| 17 | 36057890 | - | Gm11127 | NA | Stop codon | NA |
| 17 | 36081342 | - | H2-Bl | NA | Stop codon | NA |
| 17 | 36127643 | rs30755418 | Gm7030 | NA | Stop codon | NA |
| 17 | 36128930 | rs107852089 | Gm7030 | NA | Stop codon | NA |
| 17 | 36164665 | rs244535097 | Gm8909 | NA | Stop codon | NA |
| 17 | 36256003 | - | Rpp21 | NA | Stop codon | NA |
| 17 | 36936244 | rs33276926 | 1700031A10Rik | 2.62 | GAM | NA |
| 17 | 36954358 | 96711_at | Znrd1 | 1.45 | Microarray | fat |
| 17 | 37085191 | 98321_at | Olfr90 | 3.35 | Microarray | muscle |
| 17 | 37092934 | 98779_at | Olfr91 | 1.45 | Microarray | fat |
| 17 | 37770845 | rs52115539 | Olfr122 | 2.59 | GAM | NA |
| 17 | 40151125 | rs52089211 |  | 2.51 | GAM | NA |
| 17 | 40207141 | rs46497182 | Pgk2 | 2.54 | GAM | NA |
| 17 | 40225447 | rs48345817 | Crisp3 | 2.51 | GAM | NA |
| 17 | 40233028 | rs45950123 | Crisp3 | 2.85 | GAM | NA |
| 17 | 40308289 | rs49617457 | Crisp1 | 2.82 | GAM | NA |
| 17 | 40804377 | rs33695001 | Crisp2 | 2.76 | GAM | NA |
| 17 | 40804567 | rs33428427 | Crisp2 | 3.83 | GAM | NA |
| 17 | 40880220 | rs33332208 | 9130008F23Rik | NA | Stop codon | NA |
| 17 | 40880375 | rs33622016 | 9130008F23Rik | NA | Stop codon | NA |
| 17 | 40934685 | 99613_at | Mut | 2.39 | Microarray | liver |
| 17 | 40958645 | rs33117057 | Mut | 3.10 | GAM | NA |
| 17 | 40959289 | rs6204817 | Mut | 2.98 | GAM | NA |
| 17 | 40960005 | rs33816791 |  | 2.92 | GAM | NA |
| 17 | 40961474 | rs3703153 | Mut | 2.92 | GAM | NA |
| 17 | 40961746 | rs33814783 |  | 2.92 | GAM | NA |
| 17 | 40961898 | rs33814781 | Mut | 2.92 | GAM | NA |
| 17 | 42359036 | rs45818658 | 3110082D06Rik | 3.05 | GAM | NA |
| 17 | 42360164 | rs33190708 | 3110082D06Rik | 2.79 | GAM | NA |
| 17 | 42365136 | rs33497248 | 3110082D06Rik | 2.76 | GAM | NA |
| 17 | 42377562 | rs52056553 | 3110082D06Rik | 2.79 | GAM | NA |
| 17 | 42389921 | rs33359634 | 3110082D06Rik | 2.56 | GAM | NA |
| 17 | 44741564 | 94514_s_at | Arcn1 | 1.73 | Microarray | fat |
| 17 | 44741564 | 94514_s_at | Arcn1 | 3.57 | Microarray | liver |
| 17 | 46098746 | rs47101159 |  | 3.90 | GAM | NA |
| 17 | 46161032 | 104144_at | Gtpbp2 | 2.43 | Microarray | liver |
| 17 | 46251668 | rs13470593 | Yipf3 | NA | Stop codon | NA |
| 17 | 46496789 | 97179_at | BC048355 | 1.58 | Microarray | fat |
| 17 | 46646229 | 95067_at | Mrpl2 | 2.79 | Microarray | liver |
| 17 | 46646229 | 95067_at | Mrpl2 | 3.98 | Microarray | muscle |

bp=location of the gene or probe in base pair; Identifier = microarray probe (e.g., 96285_at) or rs number for genetic variants, including stop codons. Gene = official gene symbol, when relevant. Type = Microarray reflects gene expression; GAM refers to genotype association mapping. Tissue refers to the expression data from muscle, liver and adipose samples and only pertains to the microarray data. All locations are relative to GRCm38. The table is ordered by chromosome and sorted by base pair location.
